# scCRT: a contrastive-based dimensionality reduction model for scRNA-seq trajectory inference

**DOI:** 10.1093/bib/bbae204

**Published:** 2024-05-02

**Authors:** Yuchen Shi, Jian Wan, Xin Zhang, Tingting Liang, Yuyu Yin

**Affiliations:** Hangzhou Dianzi University, Hangzhou City, Zhejiang Province, China; Hangzhou Dianzi University, the Key Laboratory of Biomedical Intelligent Computing Technology of Zhejiang Province, and Zhejiang University of Science and Technology, Hangzhou City, Zhejiang Province, China; Hangzhou Dianzi University, Hangzhou City, Zhejiang Province, China; Hangzhou Dianzi University, Hangzhou City, Zhejiang Province, China; Hangzhou Dianzi University, Hangzhou City, Zhejiang Province, China

**Keywords:** single–cell RNA–sequencing, trajectory inference, contrastive learning, representation learning

## Abstract

Trajectory inference is a crucial task in single-cell RNA-sequencing downstream analysis, which can reveal the dynamic processes of biological development, including cell differentiation. Dimensionality reduction is an important step in the trajectory inference process. However, most existing trajectory methods rely on cell features derived from traditional dimensionality reduction methods, such as principal component analysis and uniform manifold approximation and projection. These methods are not specifically designed for trajectory inference and fail to fully leverage prior information from upstream analysis, limiting their performance. Here, we introduce scCRT, a novel dimensionality reduction model for trajectory inference. In order to utilize prior information to learn accurate cells representation, scCRT integrates two feature learning components: a cell-level pairwise module and a cluster-level contrastive module. The cell-level module focuses on learning accurate cell representations in a reduced-dimensionality space while maintaining the cell–cell positional relationships in the original space. The cluster-level contrastive module uses prior cell state information to aggregate similar cells, preventing excessive dispersion in the low-dimensional space. Experimental findings from 54 real and 81 synthetic datasets, totaling 135 datasets, highlighted the superior performance of scCRT compared with commonly used trajectory inference methods. Additionally, an ablation study revealed that both cell-level and cluster-level modules enhance the model’s ability to learn accurate cell features, facilitating cell lineage inference. The source code of scCRT is available at https://github.com/yuchen21-web/scCRT-for-scRNA-seq.

## INTRODUCTION

The development of single-cell RNA-sequencing (scRNA-seq) technology has enabled researchers to study the dynamic process of cell differentiation by analyzing gene expression at the single-cell level [1, 2]. Conventional scRNA-seq data analysis comprises two main steps [3]. The initial step involves upstream analysis, wherein raw count expression data are denoised [4], and cells are clustered to annotate cell type or state [5, 6]. The subsequent step involves downstream analysis to interpret biological mechanisms, including cell trajectory inference and gene enrichment analysis. The reconstructed lineage of each cell state reveals the dynamical processes of cell differentiation [7]. Therefore, it is imperative to develop a reliable trajectory inference algorithm capable of accurately identifying cell lineages, particularly for researchers studying the dynamical processes of cells progressing from an early state to a developed state.

In recent years, various trajectory inference approaches have been developed for identifying cell lineages. Based on the patterns of inferred trajectories, these algorithms can be categorized as cell-based methods or partition-based methods. Cell-based methods, such as Monocle [8], DPT [9] and scTEP [10], construct trajectories between cell points in low-dimensional space. For example, Monocle uses independent component analysis [11] to obtain cell features in low-dimensional space and builds a minimum spanning tree (MST) between cells to construct the trajectory. DPT establishes a relationship graph between cells using a weighted k-nearest-neighbor (KNN) algorithm and employs transition probabilities to reconstruct cell trajectories. scTEP applies the scDHA [12] pipeline to cluster cells and uses the results to generate a robust ensemble pseudotime. The second category, partition-based methods, divides all cells into individual partitions and connects them to construct the trajectory, with algorithms such as Monocle2 [13], TSCAN [14] and PAGA [15] falling into this category. For instance, Monocle2 uses reverse graph embedding [16] to partition cells, learns explicit principal graphs based on the partitions while reducing cell dimensionality and infers trajectories according to these partitions. This approach avoids the problems associated with complex structures and time-consuming issues encountered when inferring trajectories based on all cells in Monocle [10]. TSCAN considers labeled cell types as partitions and builds an MST between the centers of each cell type following dimensionality reduction to infer cell lineages. PAGA uses the Louvain algorithm [17] to divide cells into partitions or directly employs cell clusters and calculates connection probabilities, connecting partitions with probabilities exceeding a specific threshold.

Despite the progress in trajectory inference approaches, reconstructing cell lineages remains challenging, as existing methods focus on improving trajectory inference without optimizing dimensionality reduction methods. Dimensionality reduction, clustering analysis and trajectory inference pose major challenges in scRNA-seq analysis [18, 19]. In particular, the impact of dimensionality reduction on the accuracy of cluster and trajectory analysis is crucial. Traditional dimensionality reduction methods, including principal component analysis (PCA) and the uniform manifold approximation and projection (UMAP) [20] method, are not specifically designed for scRNA-seq data. Therefore, numerous researchers have attempted to design dimensionality reduction methods tailored for single-cell data. With the rapid growth of deep learning, neural networks have exhibited significantly superior feature learning capabilities compared with traditional methods and have demonstrated its promising potential in the analysis task within the field of bioinformatics [21]. In scRNA-seq clustering task, the neural networks are usually applied to learn cell features rather than traditional dimensionality reduction methods. For instance, scDeepCluster [22] employs the deep autoencoder, a neural network used to feed an input into the encoder to extract features and subsequently feed it into a decoder to reconstruct the original input space [23], to learn cell features and enhance clustering. Additionally, scGNN [24] incorporates a graph convolutional network [25] to train cell features, and scGAC [26] introduces a graph attention network [27] to aggregate cell representations based on between-cell importance. However, the progress of these methods is limited, as unsupervised learning patterns pose challenges for deep learning models in learning accurate cell features [28].

Contrastive learning, with its self-supervised pattern, shows promise in overcoming the aforementioned limitations [29, 30]. With its widespread application in assisting models to extract more discriminative feature space in bioinformatics [31], contrastive learning is gradually being introduced to scRNA-seq data to learn cell representations. For example, both scGCL [32] and CL-Impute [33] use contrastive learning to improve imputation of scRNA-seq upstream analysis by learning trustworthy cell representations. Additionally, scDCCA [34], CLEAR [28] and scCCL [35] enhance clustering by introducing contrastive learning to learn cell features. Although these contrastive learning-based methods achieve superior performance in scRNA-seq analysis, there are few methods that introduce deep networks to learn cell features in trajectory tasks. In a word, existing trajectory methods neglect the improvement of dimensionality reduction methods and fail to fully use prior information, such as identified cell states, when learning cell features. Based on the above observation, it is essential to design a dimensionality reduction method specifically for identifying the trajectory to improve the accuracy of the trajectory inference task.

Here, we introduce scCRT, a novel dimensional reduction model that utilizes **C**ontrastive learning to learn cell **R**epresentation to facilitate **sc**RNA-seq **T**rajectory inference. scCRT employs two feature learning components, a cell-level pairwise module and a cluster-level contrastive module, to learn accurate positional representations of cells conducive to inferring cell lineages. Initially, scRNA-seq expression data are mapped to a reduced-dimensionality space using a two-layer feedforward neural network (FNN). Then the two modules of scCRT optimize cell representations simultaneously. Specifically, the cell-level pairwise module selects one positive and one negative cell for each cell based on the distance in the original space in each training epoch. Then the module minimizes the distance of positive cells in the reduced-dimensionality space while maximizing the distance of corresponding negative cells. Meanwhile, the cluster-level module constructs two augmentation clusters for each cell cluster based on the provided cell-type labels. Then the module performs contrastive learning in the augmentation clusters to maximize the similarity of positive cluster pairs and minimize the similarity of all other negative pairs. In general, the cell-level pairwise module maintains cell–cell positional relationships in the original dimension while the cluster-level contrastive module prevents cells from dispersing excessively. Finally, cell lineages are inferred based on the learned features. Benchmark experiments on 54 real and 81 synthetic datasets, totaling 135 datasets, reveal that our method outperforms the other scRNA-seq trajectory inference methods. Additionally, an ablation study demonstrates that both cell-level and cluster-level modules contribute to learning cell features that facilitate trajectory inference.

The contributions of this paper can be summarized as follows:

(1) We propose a novel method for trajectory inference that focuses on dimensionality reduction rather than inference and can utilize prior information, such as identified cell states, obtained from upstream analysis, to learn cell features.
(2) The proposed method leverages a cell-level pairwise module to learn cell–cell positional relationships from the cell-perspective, while a cluster-level contrastive module utilizes prior information to aggregate similar cells from the cluster-perspective.
(3) We conduct the proposed scCRT on 54 real and 81 synthetic trajectory datasets, and the experiments demonstrate the superior performance of scCRT compared with other methods.

## MATERIALS AND METHODS

**Figure 1:**
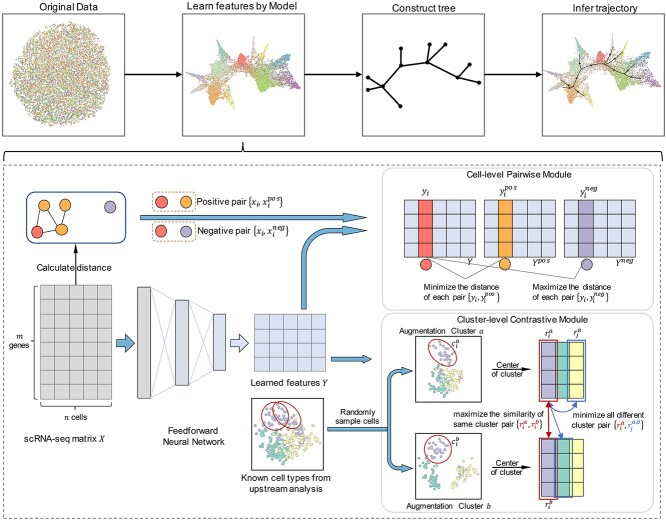
Overall architecture of scCRT. The model takes scRNA-seq data as input, and the cell-level pairwise module learns the positional representations of cells in the original space while the cluster-level contrastive module brings similar cells closer together to prevent excessive dispersion. Finally, an MST is constructed based on the learned representations, and cell lineages are inferred from the tree.

The primary architecture of scCRT, shown in Figure 1, consists of two major components: the cell-level pairwise feature learning module and the cluster-level contrastive learning module. scCRT takes the scRNA-seq count matrix $X$ as input and outputs the cell features $Y$. Then an MST is constructed based on the learned features $Y$, and cell lineages are inferred in this tree.

### Notation definition

Formally, we denote a preprocessed scRNA-seq matrix $X\in R^{n\times m}$, with $n$ cells and $m$ genes, and an identified cell type $C\in R^{n}$, which represents the cluster to which each cell belongs. The model learns the cell features $Y\in R^{n\times d}$, where $d$ is the feature size. In this process, the cell-level module creates a positive pair $\{x_{i}, x^{pos}_{i}\}$ and negative pair $\{x_{i}, x^{neg}_{i}\}$ from each cell $x_{i}\in X$. And the cluster-level module generates two augmentation clusters $c^{a}_{i}$ and $c^{b}_{i}$ from each cluster $c_{i}$ for contrastive learning, where $c_{i}=\{x^{i}_{1}, x^{i}_{2},\cdots ,x^{i}_{|c_{i}|}\}$ represents all cells belonging to cluster $i$, and $c^{a}_{i}$ and $c^{b}_{i}$ are subsets of $c_{i}$.

### Cell-level feature learning module

Most existing trajectory inference methods construct an MST based on dimensionality-reduced cell features. Therefore, precise cell-feature learning plays an important role in improving trajectory inference accuracy. The proposed cell-level module learns cell features in a reduced-dimensionality space while preserving cell–cell positional relationships in the original space. Specifically, a positive and a negative pair are constructed for each cell based on cell distance. The task of the cell-level module is to minimize the distance of each positive cell pair in the low-dimensional space while maximizing the distance of each negative pair.

Given the processed scRNA-seq count matrix $X_{n\times m}$ as input, we initially adopt the Euclidean distance to calculate a distance matrix between all cells, thereby determining positive and negative samples for each cell in each training epoch. In brief, the distance between cell $i$ and $j$ can be described by the following formula: 


(1) \begin{align*}& d_{i,j}=\sqrt{\sum^{m}_{k=1}\left(x_{i,k}-x_{j,k}\right)^{2}},\end{align*}

where $x_{i,k}$ and $x_{j,k}$ represent the $k$-th gene expression values of cells $i$ and $j$ in $X_{n\times m}$, respectively. A certain number of cells nearest to cell $i$ are selected as its positive set $P_{i}$, whereas a certain number of cells furthest from cell $i$ form its negative sample set $N_{i}$.

For each cell $i$, the model randomly selects a cell from its positive set $P_{i}$ to form pair data $\{x_{i}, x^{pos}_{i}\}$ and a cell from $N_{i}$ to form negative data $\{x_{i}, x^{neg}_{i}\}$. This operation creates the cell data $\{x_{1}, x_{2},\cdots ,x_{n}\}$ as well as its positive set $\{x^{pos}_{1}, x^{pos}_{2},\cdots ,x^{pos}_{n}\}$ and negative set $\{x^{neg}_{1}, x^{neg}_{2},\cdots ,x^{neg}_{n}\}$ as inputs. A two-layer FNN is then used to learn the features $Y$ of $X$ in low-dimensional space via $y_{i}=\mathrm{FNN}(x_{i})$. The Euclidean function is applied to measure the distance between positive and negative pairs in this low-dimensional space: 


(2) \begin{gather*} dis^{pos}_{i} = \sqrt{\sum^{d}_{k=1}\left(y_{i,k}-y^{pos}_{i,k}\right)^{2}}, \end{gather*}

(3) \begin{gather*} dis^{neg}_{i} = \sqrt{\sum^{d}_{k=1}\left(y_{i,k}-y^{neg}_{i,k}\right)^{2}}, \end{gather*}

where $d$ is the feature size of the cell in low-dimensional space. To maintain the cells’ positional relationship of the original dimension in this low-dimensional space, we optimize each cell $i$ using cross-entropy loss to bring the distance of each cell closer to its positive cell and further away from its negative cell: 


(4) \begin{align*}& {loss}_{i} = -\mathrm{log}\frac{\mathrm{exp}(dis^{pos}_{i})}{\mathrm{exp}(dis^{pos}_{i}) + \mathrm{exp}(dis^{neg}_{i})}.\end{align*}

Finally, the loss of the cell-level module is described as follows: 


(5) \begin{align*}& L_{cell} = \sum^{n}_{i=1}{loss}_{i},\end{align*}

where $n$ denotes the number of cells.

### Cluster-level contrastive learning module

Although the cell-level module can learn the cell positional representations in the reduced-dimensionality space, the learned representations may be too scattered (refer to Figure 4(B) in section 3.3). And these overly scattered cell representations may have a negative impact on trajectory inference. Therefore, a cluster-level contrastive module is introduced to bring similar cells closer together. Drawing inspiration from previous studies on contrastive learning [36–38], we construct augmentation cluster pairs to maximize the similarity of cells with the same cell states while minimizing different cells.

Given all input cells $\{x_{1}, x_{2},\cdots ,x_{n}\}$ and the cell types, $C$, to which each cell belongs, we perform data augmentation operation to generate augmentation clusters. Formally, a certain proportion of cells are randomly sampled twice from each cluster $c_{i}=\{x^{i}_{1}, x^{i}_{2},\cdots ,x^{i}_{|c_{i}|}\}$, where $x^{i}$ represents the cell $x$ belong to cluster $i$. This gives two augmented clusters, $c^{a}_{i}=\{x^{i,a}_{1}, x^{i,a}_{2},\cdots ,x^{i,a}_{|c^{a}_{i}|}\}$ and $c^{b}_{i}=\{x^{i,b}_{1}, x^{i,b}_{2},\cdots ,x^{i,b}_{|c^{b}_{i}|}\}$, for each cluster $i$, where $c_{i}$ denotes cells belonging to cluster $i$ and $|c^{a}_{i}|=|c^{b}_{i}|=\gamma |c_{i}|$ and $\gamma $ denotes the sampling ratio. The two generated augmented clusters from the same cluster are considered as a positive pair $\{c^{a}_{i}, c^{b}_{i}\}$, whereas the others, $\{c^{a}_{i}, c^{b}_{j}\}$ or $\{c^{a}_{i}, c^{a}_{j}\},i\ne j$, are considered as negative pairs. Similar to the cell-level module, a two-layer FNN is used to learn the features $Y$ of $X$ via $y_{i}=\mathrm{FNN}(x_{i})$. The center of each augmented cluster is calculated by averaging the operation to represent its position in this low-dimensional space: 


(6) \begin{align*}& r^{a}_{i} = \mathrm{Mean}(c^{a}_{i})=\mathrm{Mean}\left(\{y^{i,a}_{1}, y^{i,a}_{2},\cdots,y^{i,a}_{|c^{a}_{i}|}\}\right),\end{align*}

where $r^{a}_{i}\in R^{d}$ and $d$ represents the hidden size. The same operation is applied to obtain $r^{b}_{i}$.

To aggregate cells belonging to the same cell type, cosine similarity is used to measure the distance between two augmentation clusters. The following loss function is applied to maximize the similarity of positive pairs while minimizing the similarity of negative pairs: 


(7) \begin{align*}& {loss}^{a}_{i}=-\mathrm{log}\frac{\mathrm{exp}\left(\mathrm{s}(r^{a}_{i}, r^{b}_{i})\right)}{\sum^{c}_{j=1}\big[\mathrm{exp}(\mathrm{s}(r^{a}_{i}, r^{a}_{j}))+\mathrm{exp}(\mathrm{s}(r^{a}_{i}, r^{b}_{j}))\big]},\end{align*}

where $c$ represents the number of cell states that require lineage inference, and $\mathrm{s}(x, y)=(x^{\mathrm{T}}y)$ represents the pairwise cosine similarity. The loss $\mathrm{loss}^{b}_{i}$ of the other augmentation cluster can be calculated using the same equation. Finally, the cluster loss is defined as follows: 


(8) \begin{align*}& L_{cluster} = \frac{1}{2c}\sum^{c}_{i=1}({loss}^{a}_{i} + {loss}^{b}_{i}).\end{align*}

Overall, the total loss to optimize the model is given as follows: 


(9) \begin{align*}& Loss = L_{cell} + \lambda L_{cluster},\end{align*}

where $\lambda $ denotes a hyperparameter that controls the softness of the loss.

### Trajectory inference

Upon completion of the training process, the cell lineages are inferred based on the cell features $Y$, which is the output from the model. In brief, an MST is constructed based on the KNNs between each cell type in the model’s reduced-dimensionality space. Given an initial cell type, the cell lineages are inferred based on the MST. Finally, the principal curve [39] is used to fit a smooth lineage for each branch to estimate cell pseudotime.

A number of KNNs are used to evaluate the probability connections between each cell type, similar to the work of [40]. In particular, KNNs are selected based on the Euclidean distance between each cell feature as follows: 


(10) \begin{align*}& {dis}_{i,j}=\sqrt{\sum^{d}_{k=1}(y_{i,k}-y_{j,k})^{2}},\end{align*}

where ${dis}_{i,j}$ denotes the distance of cell $j$ to cell $i$. Subsequently, an adjacency matrix $A_{n\times n}$ is constructed, setting the $k$ value of cell $j$ nearest to cell $i$ as $A_{i,j}=1$ and the others as $A_{i,j}=0$. The connection probability between each cell type is estimated based on the adjacency matrix as follows: 


(11) \begin{align*}& B_{i,j}=\frac{\sum A_{a, b}}{\left(\big||c_{i}|-|c_{j}|\big|+1\right)^{\tau}},\end{align*}

where $a\in c_{i}$ and $b\in c_{j}$. The $B_{i,j}$ represents the calculated connection probability of cluster $j$ to $i$. The hyperparameter $\tau $ in $(||c_{i}|-|c_{j}||+1)^{\tau }$ controls softness and prevents cell types with many cells from achieving a high probability of connection to all cell types.

Finally, an MST is constructed based on the connection probability matrix $B_{i,j}$. Given a starting cluster as the root node, the node orders, created by tracing a path to the leaf from the root in the tree, are considered as the cell lineages [14, 41, 42]. For pseudotime estimation, simultaneous principal curves [42] are used to concurrently fit the principal curves of multiple branching lineages and assign pseudotime values to the cells.

### Experimental details

#### Baselines

Eight commonly used or state-of-the-art methods for scRNA-seq trajectory inference were adopted as baselines, including scTite [43], scShaper [44], Slingshot [42], Monocle3 [45], scTEP [10], Totem [46], PAGA [15] and TSCAN [14]. The parameter settings for these baselines followed their provided tutorials. Prior information regarding cell type was provided to each method in the experiments, except for scShaper and Monocle3.

#### Implementation details

First, given the raw counts scRNA-seq data, we utilize the filter_genes_dispersion function from scanpy package to select the top 2000 genes and normalize them to obtain the preprocessed matrix $X$. The proposed scCRT takes $X_{n\times m}$ and the cell type $C$ as input and outputs the cell features $Y_{n\times d}$. Finally, an MST between clusters is constructed based on $Y_{n\times d}$. The trajectory $T$ is created from the MST with the given starting cluster. The inferred trajectory $T$ is used to compare with the label to calculate the evaluation metrics. In the model, the hidden sizes in the two-layer FNN are set to 128 and 16 (64 and 8 when the number of cells less than 1000). Therefore, the feature size $d$ of cells is 16 or 8. The selected nearest neighbors $k$ in the cell-level module is set to 20 or 10. The random sampling ratio $\gamma $ is 30%. In the trajectory inference process, the $k$ in is 20 and the hyperparameter $\tau $ is set to 0 (0.5 when the proportion of a cluster cells reaches more than half of the total). The hyperparameter $\lambda $ is set to 1. We adopt the adam optimizer to optimize the model parameters and set the learning rate to 1e-3.

#### Datasets

To evaluate the performance of the proposed method, 54 real and 81 synthetic datasets, totaling 135 datasets, were used for experiments (refer to the details in the Supplementary S2 Tables 1 and 5). These datasets were provided by the dynverse [47] project. The number of cells ranged from dozens to over 10 000, with cell types ranging from a few to a dozen. The datasets also included linear and nonlinear multiple branching trajectories with real cell lineages. Moreover, the real datasets were generated using different protocols including smart-seq [48, 49], Drop-seq45 [50] and 10$\times $ Chromium [51]. The synthetic datasets were simulated using dyntoy [47], prosstt [52] and splattersimulators [53], with each cell assigned distinct pseudotime values to individual cells in the order of trajectory. For the baseline experiment, the unprocessed raw counts matrix $R$ was used as input and preprocessed using the function in each method’s tutorial (refer to the Supplementary S1 Table 3 for details). In our experiment, the scanpy [54, 55] package was used to select 2000 variable genes and normalize the counts matrix for preprocessing.

#### Evaluation metrics

To assess the performance of the proposed method and the baseline methods, we followed the dynbenchmark [47] framework, using the HIM score, F1 branches and F1 milestones to measure the similarity between inferred trajectories and the real of the datasets. The HIM score measures the similarity of the predicted topologies of the trajectory and real values, although it cannot evaluate the order of cell states in the predicted topology. The F1 branches and F1 milestones measure the order of cells on the branches or milestones (center of cell states) of the trajectory, respectively. Additionally, given that each cell in the synthetic dataset had its own pseudotime values, we followed [19, 44], using the Pearson correlation coefficient (PCC) $corr(T, T^{\prime})$ to measure the similarity between the estimated pseudotime $T^{\prime}$ determined via each method and its real value $T$ in the synthetic dataset.

## RESULTS

### scCRT exhibits excellent performance on real datasets

**Figure 2:**
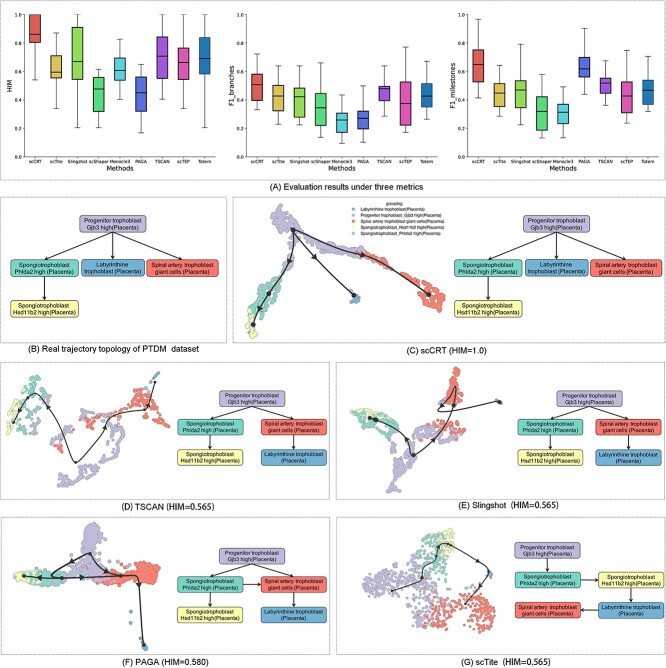
Ablation experiment results based on real and synthetic datasets. (A) Average metrics of scCRT and three ablation methods based on real and synthetic datasets. (B) Visualization of cell representation and inferred trajectory results for each method based on the multifurcating_8 dataset. (C) Evolution of the learned cell representation across the training process.

To evaluate the scCRT’s trajectory inference performance with scRNA-seq data, we conducted experiments on 54 real datasets, comparing our model with eight baseline methods. Three metrics (HIM, F1-branches and F1-milestones) were used to determine the accuracy of each method’s inferred cell trajectories. As shown by the boxplots in Figure 2(A), representing 24 real nonlinear bifurcation datasets (the results of all 54 real datasets are shown in the Supplementary S1 Table 2 and S2 Tables 2–4), scTite, Slingshot, TSCAN, scTEP and Totem performed well in all three metrics. In contrast, scShaper and Monocle got unsatisfactory results, possibly due to scShaper’s inadequate performance with nonlinear datasets and Monocle inferring trajectory based on individual cells rather than clusters. PAGA showed mixed results, exceeding in F1-milestones but performing poorly in HIM and F1-branches, likely because PAGA infers all possible paths during the trajectory inference, resulting in discrepancies from real trajectory topology. Notably, scCRT outperformed all baseline methods in the three metrics, indicating its superior ability to accurately infer trajectory topology and cluster order.

To provide an intuitive way to demonstrate that the learned cell features using scCRT facilitates the lineage inference, as shown in Figure 2(B)–(G), we visualized the ground truth and inferred cell lineage results of scCRT and the baseline methods (Supplementary S1 Figure 1) based on the *placenta trophoblast differentiation mca* (PTDM) [56] dataset using the dynplot [47] package. Figure 2(B) illustrates the real cell lineages of this dataset, and Figure 2(C)–(G) displays the inferred trajectories and cell features (left-side images) as well as the trajectories (right-side images). As shown in Figure 2(C), scCRT accurately inferred three cell lineages, aligning with the actual trajectory. Moreover, the position distribution of cell features learned by scCRT mirrored the real topology, aiding in accurate lineage inference. In contrast, other methods inferred incorrect lineages. For example, TSCAN and Slingshot produced only two lineages, and scTite identified only one. As shown in the left side of Figure 2(D)–(G), the *Labyrinthine trophoblast* (blue) is positioned closer to the *Spiral artery trophoblast giant cells* (red), indicating inaccurate cell features after dimensionality reduction, leading to incorrect cell lineage inference. Overall, the cell features’ position distribution achieved by scCRT is similar to the lineages of real trajectories, facilitating accurate trajectory inference.

### scCRT shows superior performance on synthetic datasets

**Figure 3:**
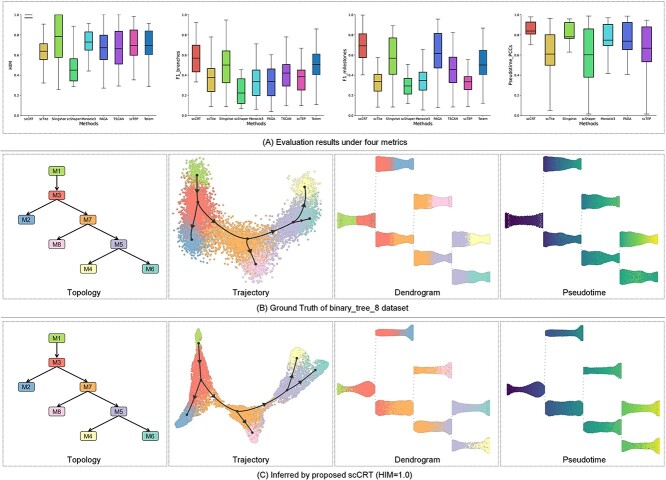
Experimental results based on 24 real nonlinear bifurcation datasets. (A) Box plots of HIM, F1-branches and F1-milestones scores based on 24 datasets for each method. The black lines within boxes represent mean scores of datasets. (B) Ground truth trajectory topology of the PTDM dataset. (C) Visualization of the inferred trajectory and its topology via scCRT. (D–G) Visualizations of results inferred via TSCAN, Slingshot, PAGA and scTite.

Estimating the cell pseudotime based on the inferred trajectory is vital for determining the developmental order of cells. We assessed whether scCRT improves the accuracy of inferred pseudotime variables on 81 synthetic datasets generated using various protocols, with each cell in the dataset exhibiting pseudotime values. The HIM, F1-branches and F1-milestones metrics were used to measure inferred cell trajectory accuracy, and PCC was employed to evaluate the similarity between estimated cell pseudotime and real values. As shown in Figure 3(A), representing 62 synthetic nonlinear bifurcation datasets (the results of all 81 datasets are shown in the Supplementary S1 Table 2 and S2 Tables 6–9), scCRT, Slingshot, PAGA, Monocle and Totem achieved satisfactory trajectory inference and pseudotime estimation, whereas scTite, scShaper and scTEP exhibited inadequate performance. scCRT consistently outperformed all baseline methods in the four metrics, achieving the best average values. Notably, the HIM metric of scCRT, close to 1 on all datasets, indicated nearly perfect consistency between inferred cell trajectories and reality. The superior PCC values confirmed scCRT’s calculated cell pseudotime being closely aligned with the actual time, likely due to trustworthy cell features learned by scCRT. Overall, scCRT exhibited superior performance in learning cell features across datasets generated using different simulators, enhancing trajectory inference and pseudotime estimation.

We also visualized the ground truth and inferred trajectory results based on the binary_tree_8 dataset, as shown in Figure 3(B) and (C), respectively (the other baseline methods are shown in the Supplementary S1 Figure 2). In this figure, the topology subfigure represents the cluster order in the trajectory, the dendrogram subfigure represents branch evolution and the corresponding positions of cells in the trajectory (color-coded by cell type) and the right pseudotime subfigure denotes the dendrogram (color-coded based on estimated pseudotime). Specifically, the results in Figure 3(C) show that the trajectory and pseudotime inferred by scCRT closely match the ground truth. In contrast, the trajectory branches inferred by other methods exhibit inaccuracies (Supplementary S1 Figure 2), leading to imprecise pseudotime estimation. Furthermore, the trajectory subfigure in Figure 3(C) reveals that the distribution of cells between different clusters aligns more consistently with the ground truth trajectory, promoting accurate trajectory inference based on these cell features. Overall, this suggests that scCRT’s distribution of cells between different clusters in the reduced-dimensionality space aligns more consistently with the cell development trajectory, facilitating accurate trajectory inference and aiding in estimating precise pseudotime values.

**Figure 4:**
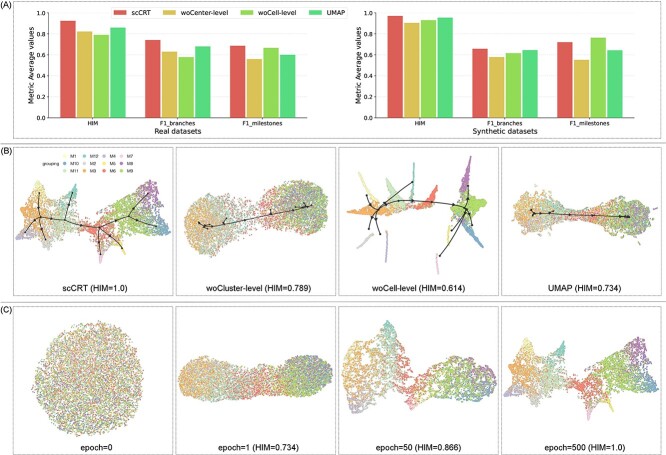
Experimental results based on 62 synthetic nonlinear bifurcation datasets. (A) Box plots of HIM, F1-branches, F1-milestones and PCC scores based on synthetic datasets for each method. The black lines within boxes represent mean scores of datasets. (B) Visualization of ground truth trajectory topology of the binary_tree_8 dataset and its dendrogram and pseudotime. (C) Visualization of the inferred trajectory and its topology, dendrogram and pseudotime achieved using scCRT.

### Ablation study

To demonstrate the effectiveness of the feature learning modules in scCRT, we performed ablation studies on the 54 real and 81 synthetic datasets. This involved removing the cell-level module (woCluster) and the cluster-level module (woCell), and using only UMAP [20] for obtaining cell features. The performance of these three ablation methods is shown in Figure 4(A). Notably, woCell outperformed the other methods for F1-milestones in synthetic datasets, indicating a higher score without necessarily implying more accurate trajectory inference. The F1-milestone score indicates proximity to the cluster center, and woCell’s superior score arise due to its reliance on the cluster-level module, causing cells to converge toward its center.

Figure 4(B) provides visualizations of each ablation method based on a multiple branching dataset. Specifically, the UMAP’s cell positions in the reduced-dimensionality space (right side of the figure) were excessively dispersed, hindering observation of cell lineages in developmental trajectories. This may be due to UMAP lacking a cluster-module that gathers cells with the same state. As shown in the middle part of Figure 4(B), the woCluster ablation method was unsatisfactory. Additionally, visualization of the woCell method revealed that cells were overly clustered, losing positional relationships between cells due to cell-level module removal. In contrast, the dimension reduction result of scCRT is shown in the left side of Figure 4(B), it can be seen that the learned cell position features fully match the shape of the trajectory. Figure 4(C) illustrates the evolution of learned cell representation during training. Initially chaotic (epoch = 0), the cells gradually organized between clusters as training epochs progressed (epoch = 1, 50), a process that prevents excessive dispersion. Ultimately, the cell distribution in the reduced-dimensionality space formed the trajectory (epoch = 500), as shown in the left side of Figure 4(B). Overall, scCRT’s superior trajectory inference performance is attributed to the cell-level and cluster-level modules, enabling simultaneously learning of positional relationships between cells and clusters in the reduced-dimensionality space.

### Gene expression analysis on a real dataset

**Figure 5:**
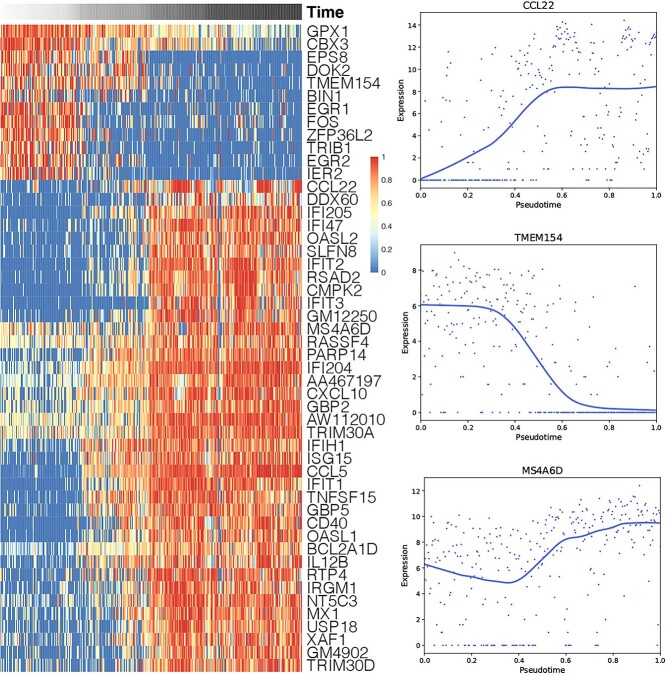
Gene expression changes related to the pseudotime of a lipopolysaccharide dataset. (Left) Heatmap of 50 key genes’ expression patterns related to the inferred trajectory. (Right) Expression changes of genes with three different expression patterns relative to the inferred cell pseudotime.

To demonstrate the scCRT’s ability to accurately recapitulate the cell pseudotime, we performed a genetic analysis on bone marrow-derived mouse dendritic cells stimulated by liposaccharide (LPS) [57]. This dataset, comprising 307 cells collected at 1, 2, 4 and 6 h, is widely used for trajectory analysis. Similar to previous research [58], we analyzed the behavior of certain genes by ordering gene expression based on the pseudotime estimated via scCRT. We visualized key genes in a heatmap (Figure 5), assessing their importance to the inferred trajectory using random forests [59]. The heatmap in Figure 5 shows that the expression levels of these important genes exhibit three distinct patterns, consistent with prior findings [58]. The first pattern involves low initial expression, followed by a gradual increase along pseudotime and high expression in postprogression, exemplified by *CCL22* (upper right subfigure in Figure 5). The second pattern involves high initial expression with a decrease in postprogression, as shown by *TMEM154* (middle right subfigure in Figure 5). The third pattern involves initial expression that decreased along pseudotime, before increasing again and achieving high expression in postprogression, as illustrated by *MS4A6D* (lower right subfigure in Figure 5). Previous studies [58] have also reported these three expression patterns, suggesting the third pattern as a switch-like pattern during stimulation. In conclusion, scCRT accurately estimates the pseudotime of the cell trajectory.

## CONCLUSION

In scRNA-seq data analysis, inferring cell lineages for trajectory reconstruction is a crucial downstream task. However, existing trajectory methods often rely on cell representations obtained through conventional dimensionality reduction methods (e.g. PCA and UMAP). These methods overly focus on trajectory inference and neglect to optimize the dimensionality reduction methods, resulting in the inability to fully utilize prior information when learning cell features. Based on these observation, we introduce a deep learning-based dimensionality reduction model, scCRT, specifically designed for trajectory analysis. This model maximizes the use of prior cell type information derived from upstream clustering tasks. scCRT excels at learning precise cell representations in a reduced-dimensionality space, preserving cell–cell positional relationships in the original space and concurrently aggregating cells of the same type to prevent excessive dispersal in the low-dimensional space. Experiments conducted on 54 real and 81 synthetic datasets highlighted the outstanding performance of the proposed model compared with commonly used or existing state-of-the-art trajectory inference methods.

In this paper, we provide a new perspective to improve the trajectory inference method, which focuses on dimension reduction rather than inference. The proposed method can utilize prior information, such as identified cell states, obtained from upstream analysis, when learning cell features. However, there are still some potential limitations in this approach. For example, the cell type is required as input for learning features. If the provided cell type information has noise, the learned features will be inaccurate. Besides, the presence of the discrete outlier cluster will affect the trajectory prediction. In future research, we will focus on reducing the negative impact caused by inaccurate prior information and refining the inference of cell lineages.

**Key Points**

- scCRT is a dimensionality reduction model for scRNA-seq trajectory inference.
- scCRT learns cell features while maintaining its positional relationships.
- scCRT aggregates cells using prior information from upstream tasks.
- scCRT outperformed other methods in a trajectory inference experiment.

## FUNDING

This work was supported by Yangtze River Delta Project [Grant 2023ZY1068], and ‘Pioneer” and “Leading Goose’ R&D Program of Zhejiang, China [Grant 2022C03043].

## DATA AVAILABILITY

The source code of scCRT is available at https://github.com/yu chen21-web/scCRT-for-scRNA-seq. Moreover, to demonstrate the authenticity of the experiment, some experimental data and a trained model have been publicly available for verifying the results of this paper.

## Supplementary Material

Supplementary_S1_bbae204

Supplementary_S2_bbae204

## Author Biographies

**Yuchen Shi** is a Ph.D candidate at Hangzhou Dianzi University, Hangzhou, China. His research interest is scRNA-seq data analysis and deep learning.

**Jian Wan** is a Professor at Hangzhou Dianzi University, the Key Laboratory ofBiomedical Intelligent Computing Technology of Zhejiang Province, and Zhejiang University of Science and Technology. His research interest is committed to deeply integrating biotechnology and information technology.

**Xin Zhang** is a Lecture at Hangzhou Dianzi University. Her research interest includes deep learning and bioinformation analysis.

**Tingting Liang** is an Associate Professor at Hangzhou Dianzi University. Her research interest is focused on deep learning.

**Yuyu Yin** is a Professor at the Key Laboratory of Complex Systems Modeling and Simulation Ministry of Education, Hangzhou Dianzi University. His laboratory is focused on complex data modeling and analysis.
