# Supplementary material for: scCRT: a contrastive-based dimensionality reduction model for scRNA-seq trajectory inference
Source: Brief Bioinform. 2024 May 2;25(3):bbae204. doi: 10.1093/bib/bbae204 (PMC11066919; doi:10.1093/bib/bbae204)

**Supplementary Table 1. Average scores of real datasets for each method.**

| Methods | All 54 real datasets | | | 24 real bifurcation datasets | | |
| --- | --- | --- | --- | --- | --- | --- |
|  | HIM | F1 branches | F1 milestones | HIM | F1 branches | F1 milestones |
| scCRT | **0.922** | **0.736** | **0.689** | **0.862** | **0.507** | **0.649** |
| scTite | 0.827 | 0.681 | 0.547 | 0.595 | 0.428 | 0.448 |
| Slingshot | 0.829 | 0.685 | 0.602 | 0.670 | 0.423 | 0.470 |
| scShaper | 0.779 | 0.723 | 0.505 | 0.477 | 0.345 | 0.320 |
| Monocle3 | 0.593 | 0.316 | 0.351 | 0.607 | 0.259 | 0.324 |
| PAGA | 0.472 | 0.368 | 0.600 | 0.451 | 0.271 | 0.618 |
| TSCAN | 0.822 | 0.694 | 0.602 | 0.708 | 0.479 | 0.518 |
| scTEP | 0.811 | 0.627 | 0.518 | 0.663 | 0.375 | 0.428 |
| Totem | 0.635 | 0.442 | 0.455 | 0.678 | 0.418 | 0.467 |

*These results are converted into the format used for evaluation in dyno project based on the output of each method, and different conversion methods may result in different results.

* dyno package is available in https://github.com/dynverse/dyno.

**Supplementary Table 2. Average scores of synthetic datasets for each method.**

| Methods | All 81 synthetic datasets | | | | 62 synthetic bifurcation datasets | | | |
| --- | --- | --- | --- | --- | --- | --- | --- | --- |
|  | HIM | F1 branches | F1 milestones | PCCs | HIM | F1 branches | F1 milestones | PCCs |
| scCRT | **0.971** | **0.658** | **0.720** | **0.828** | **0.969** | **0.568** | **0.690** | **0.838** |
| scTite | 0.728 | 0.498 | 0.428 | 0.613 | 0.633 | 0.370 | 0.334 | 0.614 |
| Slingshot | 0.829 | 0.588 | 0.618 | 0.800 | 0.785 | 0.500 | 0.567 | 0.779 |
| scShaper | 0.579 | 0.405 | 0.427 | 0.658 | 0.450 | 0.222 | 0.292 | 0.601 |
| Monocle3 | 0.689 | 0.332 | 0.360 | 0.756 | 0.729 | 0.335 | 0.347 | 0.747 |
| PAGA | 0.660 | 0.384 | 0.603 | 0.714 | 0.672 | 0.335 | 0.615 | 0.736 |
| TSCAN | 0.728 | 0.524 | 0.530 | - | 0.662 | 0.420 | 0.454 | - |
| scTEP | 0.761 | 0.500 | 0.429 | 0.696 | 0.694 | 0.384 | 0.334 | 0.668 |
| Totem | 0.740 | 0.541 | 0.552 | - | 0.695 | 0.504 | 0.500 | - |

**Supplementary Table 3. Experimental settings for each baseline.**

|  | Preprocess and running | evaluation |
| --- | --- | --- |
| scTite | # https://github.com/dblab2022/scTite  Library(scTite)  scTite_res <- sctite(data, cell_labels, start_cluster_id) | Convert output |
| PAGA | # https://scanpy-tutorials.readthedocs.io/en/latest/pbmc3k.html  follow the tutorial | Convert output |
| TSCAN | # https://github.com/zji90/TSCAN  Library(Seurat)  Library(TSCAN)  # select top 2000 genes  pbmc <- CreateSeuratObject(counts = data)  pbmc <- NormalizeData(object = pbmc, scale.factor = 1000)  pbmc <- FindVariableFeatures(object = pbmc, nfeatures = 2000)  TopGene = VariableFeatures(object = pbmc)  # start TSCAN  prodata <- preprocess(data[TopGene ])  TSCAN_res <- exprmclust(pre_infos$prodata, cluster=cell_labels) | Convert output |
| scTEP | # https://github.com/cran/scTEP  Library(scTEP)  expr_data = preprocessing(expr_data)  data_fa <- uwot::umap(expr_data%>% as.data.frame(), n_components=20) %>% as.data.frame() %>% as.matrix()  allCluster = scTEP::clustering(expr_data, seed = 1)  scDHA_res <- scDHA(data_fa, do.clus = T, gen_fil = T, ncores = 16, seed = 1) | Convert output |
| Totem | # https://github.com/elolab/Totem  Library(Totem)  sce <- SingleCellExperiment(data)  sce <- PrepareTotem(sce)  sce <- RunDimRed(sce)  sce <- RunClustering(sce)  sce <- SelectClusterings(sce)  Totem_res <- RunSmoothing(sce) | Convert output |
| Slingshot | # https://github.com/kstreet13/slingshot  library(dynwrap)  library(ti_slingshot())  slingshot_res <- infer_trajectory(data, ti_slingshot(),verbose = FALSE)  dyneval::calculate_metrics(data, slingshot_res, c( 'him', ‘F1_branches’, ‘F1_milestones’)) | |
| scShaper | # https://github.com/elolab/scshaper  library(scShaper)  scShaper_res <- infer_trajectory(data, ti_scShaper(k.range = 2:100,num.pcs = 50,span = 0.1),verbose = FALSE)  dyneval::calculate_metrics(data, scShaper_res, c( 'him', ‘F1_branches’, ‘F1_milestones’)) | |
| Monocle3 | # https://github.com/cole-trapnell-lab/monocle3  library(dynwrap)  library(monocle)  monocle_res <- infer_trajectory(data, ti_monocle(),verbose = FALSE)  dyneval::calculate_metrics(data, monocle_res, c( 'him', ‘F1_branches’, ‘F1_milestones’)) | |

* The “Convert outputs” is to converted the output of baseline to the format required for evaluating with dyno package.

* dyno package is available in https://github.com/dynverse/dyno

**Supplementary Figure 1. The visualization of ground truth and inferred cell lineages results of each method on placenta trophoblast differentiation mca dataset.**


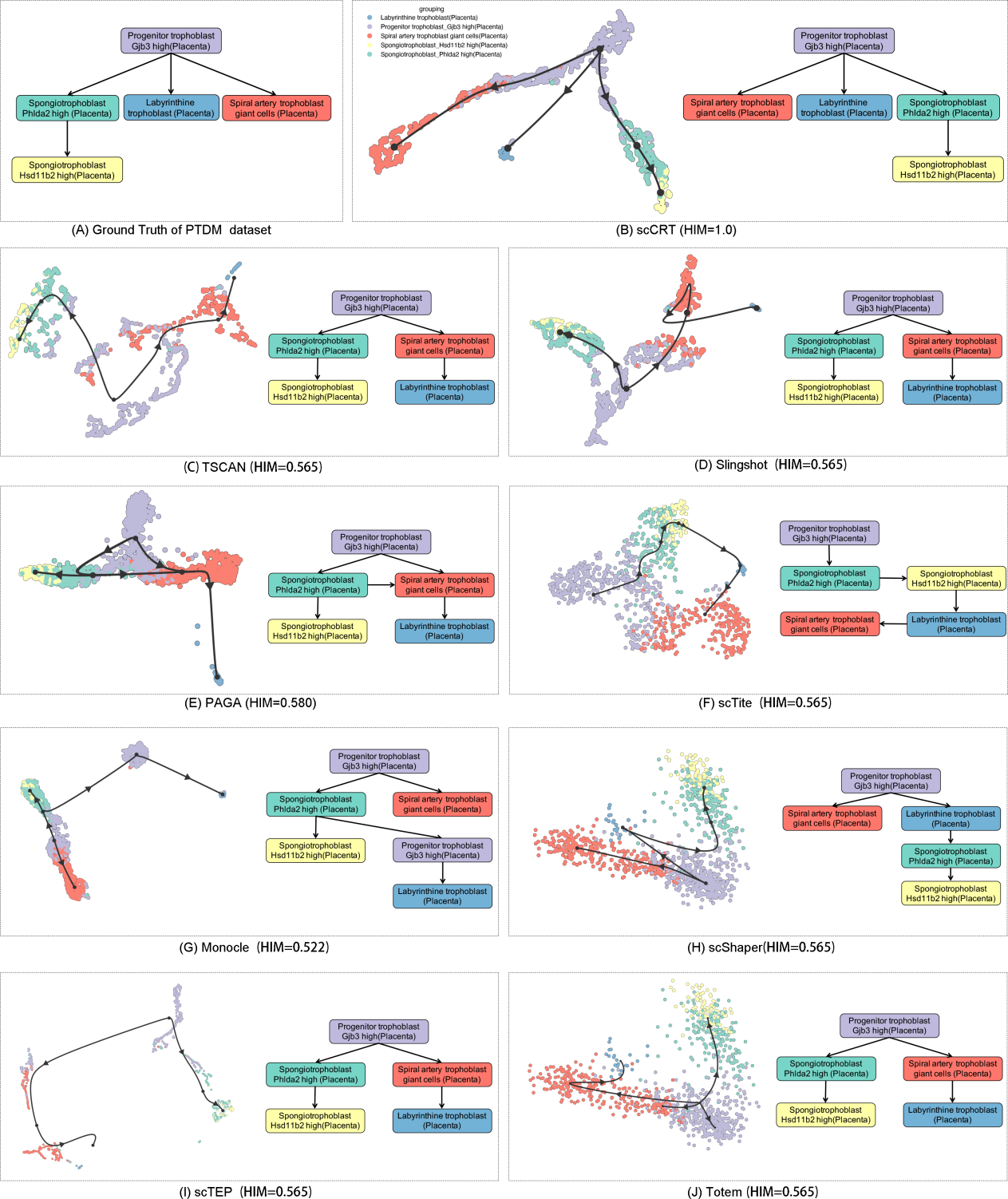


**Supplementary Figure 2. The visualization of ground truth and inferred cell lineages results of each method on binary_tree_8 dataset.**


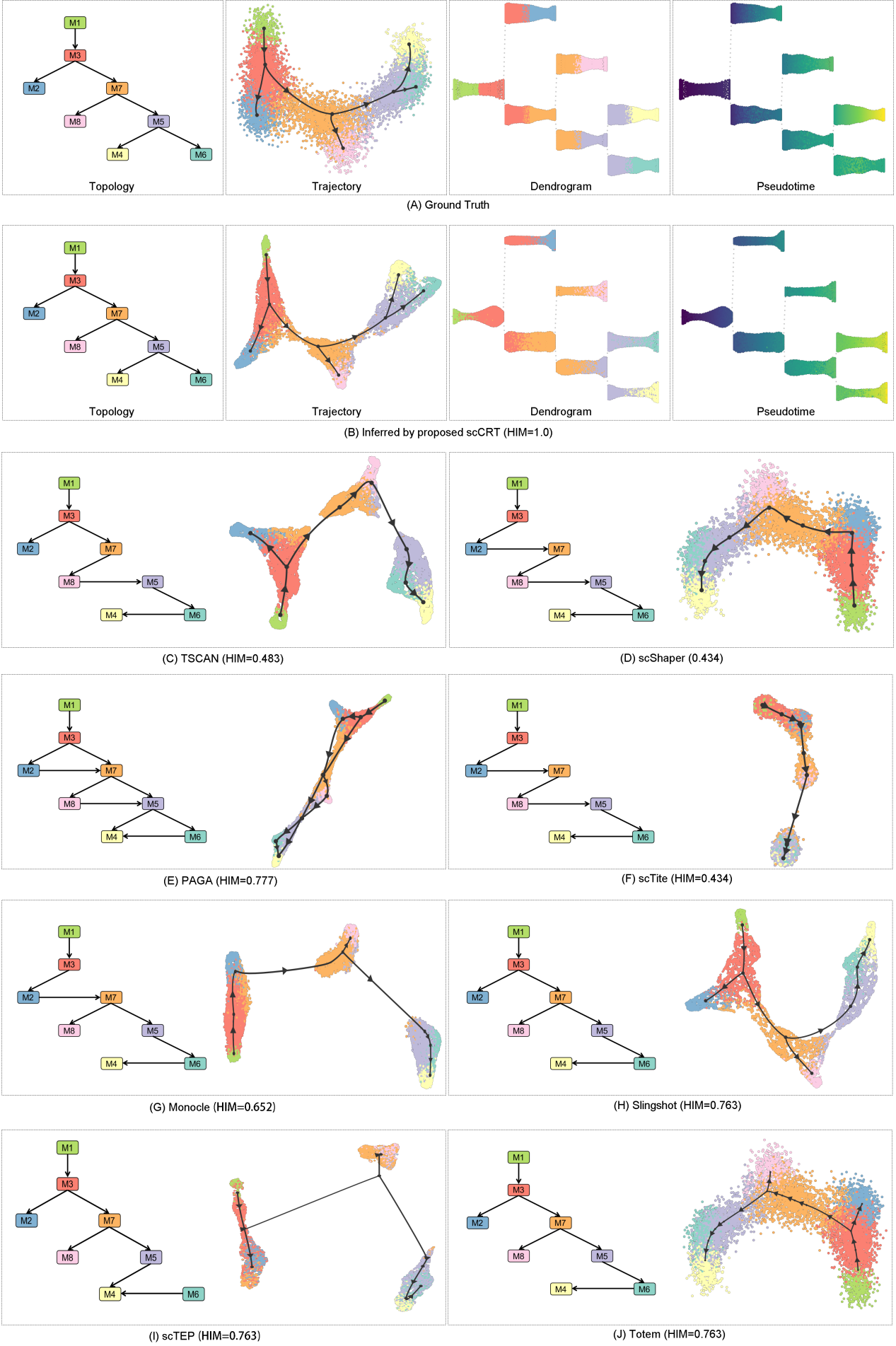

Supplement: Supplementary_S1_bbae204 [file supplementary_s1_bbae204.docx]
