# Supplementary material for: scCRT: a contrastive-based dimensionality reduction model for scRNA-seq trajectory inference
Source: Brief Bioinform. 2024 May 2;25(3):bbae204. doi: 10.1093/bib/bbae204 (PMC11066919; doi:10.1093/bib/bbae204)
Supplement: Supplementary_S2_bbae204 [file supplementary_s2_bbae204.docx]

**Table 1. Details of real datasets**

|  | types | dataset_name | cell_nums | trajectory_type |
| --- | --- | --- | --- | --- |
| 1 | real-gold | aging-hsc-young_kowalczyk | 493 | linear |
| 2 | real-gold | NKT-differentiation_engel | 197 | multifurcation |
| 3 | real-gold | pancreatic-beta-cell-maturation_zhang | 562 | linear |
| 4 | real-gold | stimulated-dendritic-cells-PIC_shalek | 433 | linear |
| 5 | real-gold | myoblast-differentiation_trapnell | 290 | linear |
| 6 | real-gold | hematopoiesis-gates_olsson | 316 | linear |
| 7 | real-gold | developing-dendritic-cells_schlitzer | 238 | linear |
| 8 | real-gold | mESC-differentiation_hayashi | 414 | linear |
| 9 | real-gold | psc-astrocyte-maturation-neuron_sloan | 192 | linear |
| 10 | real-gold | germline-human-both_guo | 272 | bifurcation |
| 11 | real-gold | macrophage-salmonella_saliba | 60 | multifurcation |
| 12 | real-gold | psc-astrocyte-maturation-glia_sloan | 455 | linear |
| 13 | real-gold | aging-hsc-old_kowalczyk | 873 | linear |
| 14 | real-gold | stimulated-dendritic-cells-LPS_shalek | 540 | linear |
| 15 | real-gold | pancreatic-alpha-cell-maturation_zhang | 322 | linear |
| 16 | real-gold | stimulated-dendritic-cells-PAM_shalek | 407 | linear |
| 17 | real-gold | human-embryos_petropoulos | 1,289 | linear |
| 18 | real-silver | trophectoderm-monkey_nakamura | 82 | linear |
| 19 | real-silver | planaria-pair-4_plass | 8,960 | tree |
| 20 | real-silver | fibroblast-reprogramming_treutlein | 355 | bifurcation |
| 21 | real-silver | planaria-pharynx-differentiation_plass | 292 | linear |
| 22 | real-silver | embronic-mesenchyme-neuron-differentiation_mca | 481 | tree |
| 23 | real-silver | planaria-pair-2_plass | 10,653 | tree |
| 24 | real-silver | epidermis-hair-IFE_joost | 706 | linear |
| 25 | real-silver | planaria-pair-1_plass | 10,578 | bifurcation |
| 26 | real-silver | olfactory-projection-neurons-DA1_horns | 277 | linear |
| 27 | real-silver | oligodendrocyte-differentiation-clusters_marques | 3,685 | linear |
| 28 | real-silver | neonatal-inner-ear-TEC-SC_burns | 85 | linear |
| 29 | real-silver | planaria-pair-3_plass | 8,589 | tree |
| 30 | real-silver | neonatal-inner-ear-SC-HC_burns | 117 | linear |
| 31 | real-silver | planaria-pair-14_plass | 13,281 | tree |
| 32 | real-silver | oligodendrocyte-differentiation-subclusters_marques | 4,930 | multifurcation |
| 33 | real-silver | germline-human-female_li | 621 | linear |
| 34 | real-silver | epidermis-hair-spatial_joost | 763 | linear |
| 35 | real-silver | epidermis-hair-uHF_joost | 350 | linear |
| 36 | real-silver | planaria-epidermis-differentiation_plass | 4,003 | linear |
| 37 | real-silver | cortical-interneuron-differentiation_frazer | 213 | multifurcation |
| 38 | real-silver | thymus-t-cell-differentiation_mca | 1,607 | bifurcation |
| 39 | real-silver | planaria-muscle-differentiation_plass | 2,338 | bifurcation |
| 40 | real-silver | hematopoiesis-clusters_olsson | 376 | tree |
| 41 | real-silver | planaria-pair-8_plass | 9,354 | tree |
| 42 | real-silver | hepatoblast-differentiation_yang | 504 | bifurcation |
| 43 | real-silver | distal-lung-epithelium_treutlein | 59 | bifurcation |
| 44 | real-silver | bone-marrow-mesenchyme-erythrocyte-differentiation_mca | 3,105 | linear |
| 45 | real-silver | planaria-phagocyte-differentiation_plass | 694 | linear |
| 46 | real-silver | germline-human-male_li | 649 | linear |
| 47 | real-silver | dentate-gyrus-neurogenesis_hochgerner | 3,585 | linear |
| 48 | real-silver | planaria-pair-10_plass | 14,636 | tree |
| 49 | real-silver | planaria-pair-9_plass | 11,319 | tree |
| 50 | real-silver | planaria-pair-11_plass | 12,978 | tree |
| 51 | real-silver | neonatal-inner-ear-TEC-HSC_burns | 85 | linear |
| 52 | real-silver | planaria-neuron-differentiation_plass | 2,349 | multifurcation |
| 53 | real-silver | placenta-trophoblast-differentiation_mca | 1,001 | multifurcation |
| 54 | real-silver | planaria-parenchyme-differentiation_plass | 1,986 | multifurcation |

**Table 2. HIM of real datasets**

|  |  | HIM | | | | | | | | |
| --- | --- | --- | --- | --- | --- | --- | --- | --- | --- | --- |
|  | dataset_name | scCRT | scTite | Slingshot | scShaper | Monocle3 | PAGA | TSCAN | scTEP | Totem |
| 1 | aging-hsc-young_kowalczyk | 1.000 | 1.000 | 1.000 | 1.000 | 1.000 | 0.322 | 1.000 | 1.000 | 1.000 |
| 2 | NKT-differentiation_engel | 0.921 | 0.555 | 0.921 | 0.555 | 0.561 | 0.272 | 0.555 | 0.555 | 0.678 |
| 3 | pancreatic-beta-cell-maturation_zhang | 1.000 | 0.729 | 1.000 | 1.000 | 0.481 | 0.218 | 1.000 | 1.000 | 0.579 |
| 4 | stimulated-dendritic-cells-PIC_shalek | 0.565 | 0.584 | 0.553 | 1.000 | 0.458 | 0.268 | 0.568 | 0.730 | 0.421 |
| 5 | myoblast-differentiation_trapnell | 1.000 | 1.000 | 1.000 | 1.000 | 0.679 | 0.330 | 1.000 | 0.565 | 0.586 |
| 6 | hematopoiesis-gates_olsson | 1.000 | 1.000 | 1.000 | 1.000 | 0.652 | 0.322 | 1.000 | 1.000 | 0.407 |
| 7 | developing-dendritic-cells_schlitzer | 1.000 | 1.000 | 1.000 | 1.000 | 0.439 | 0.322 | 1.000 | 1.000 | 1.000 |
| 8 | mESC-differentiation_hayashi | 1.000 | 0.559 | 1.000 | 1.000 | 1.000 | 1.000 | 1.000 | 1.000 | 0.671 |
| 9 | psc-astrocyte-maturation-neuron_sloan | 1.000 | 1.000 | 1.000 | 1.000 | 0.477 | 0.330 | 1.000 | 1.000 | 0.399 |
| 10 | germline-human-both_guo | 0.806 | 0.559 | 0.610 | 0.559 | 0.500 | 0.269 | 0.902 | 0.687 | 0.571 |
| 11 | macrophage-salmonella_saliba | 0.921 | 0.555 | 0.921 |  | 0.555 | 0.272 | 0.921 | 0.555 | 0.555 |
| 12 | psc-astrocyte-maturation-glia_sloan | 1.000 | 1.000 | 0.560 | 1.000 | 0.542 | 0.397 | 0.560 | 0.567 | 0.288 |
| 13 | aging-hsc-old_kowalczyk | 1.000 | 1.000 | 1.000 | 1.000 | 0.601 | 0.322 | 1.000 | 1.000 | 1.000 |
| 14 | stimulated-dendritic-cells-LPS_shalek | 1.000 | 1.000 | 1.000 | 1.000 | 0.368 | 0.318 | 0.568 | 1.000 | 1.000 |
| 15 | pancreatic-alpha-cell-maturation_zhang | 1.000 | 1.000 | 1.000 | 1.000 | 0.651 | 0.193 | 1.000 | 0.667 | 0.488 |
| 16 | stimulated-dendritic-cells-PAM_shalek | 1.000 | 1.000 | 1.000 | 1.000 | 0.387 | 0.311 | 1.000 | 1.000 | 0.562 |
| 17 | human-embryos_petropoulos | 1.000 | 1.000 | 1.000 | 1.000 | 0.382 | 1.000 | 1.000 |  | 0.435 |
| 18 | trophectoderm-monkey_nakamura | 1.000 | 1.000 | 1.000 | 1.000 | 0.562 | 1.000 | 1.000 | 1.000 | 0.423 |
| 19 | planaria-pair-4_plass | 0.808 |  | 0.430 | 0.226 | 0.653 | 0.555 | 0.405 | 0.529 |  |
| 20 | fibroblast-reprogramming_treutlein | 0.801 | 0.617 | 1.000 | 0.508 | 0.569 | 0.399 | 1.000 | 0.606 | 0.735 |
| 21 | planaria-pharynx-differentiation_plass | 1.000 |  | 1.000 | 1.000 | 0.669 | 1.000 | 1.000 |  | 0.350 |
| 22 | embronic-mesenchyme-neuron-differentiation_mca | 0.943 | 0.715 | 0.387 | 0.387 | 0.632 | 0.424 | 0.747 | 0.721 | 0.676 |
| 23 | planaria-pair-2_plass | 0.747 |  | 0.545 | 0.218 | 0.703 | 0.614 | 0.709 | 0.599 |  |
| 24 | epidermis-hair-IFE_joost | 1.000 | 1.000 | 1.000 | 1.000 | 0.616 | 0.396 | 1.000 | 1.000 | 0.567 |
| 25 | planaria-pair-1_plass | 0.413 |  | 0.391 | 1.000 | 0.403 | 0.169 | 0.490 | 0.447 |  |
| 26 | olfactory-projection-neurons-DA1_horns | 1.000 | 1.000 | 1.000 | 1.000 | 0.605 | 0.574 | 0.565 | 1.000 | 0.568 |
| 27 | oligodendrocyte-differentiation-clusters_marques | 0.560 |  | 1.000 | 1.000 | 0.424 | 0.322 | 1.000 | 1.000 | 1.000 |
| 28 | neonatal-inner-ear-TEC-SC_burns | 1.000 | 1.000 | 1.000 | 1.000 | 0.701 | 0.322 | 1.000 | 1.000 | 1.000 |
| 29 | planaria-pair-3_plass | 1.000 |  | 0.546 | 0.317 | 0.693 | 0.630 | 0.749 | 0.696 |  |
| 30 | neonatal-inner-ear-SC-HC_burns | 1.000 | 1.000 | 1.000 | 1.000 | 1.000 | 0.498 | 1.000 | 1.000 | 0.390 |
| 31 | planaria-pair-14_plass | 0.886 |  | 0.737 | 0.332 | 0.704 | 0.553 | 0.806 | 0.867 |  |
| 32 | oligodendrocyte-differentiation-subclusters_marques | 0.686 |  | 0.686 | 0.269 | 0.693 | 0.524 | 0.705 | 0.400 |  |
| 33 | germline-human-female_li | 1.000 |  | 1.000 | 1.000 | 0.522 | 1.000 | 1.000 | 1.000 | 0.342 |
| 34 | epidermis-hair-spatial_joost | 1.000 | 1.000 | 1.000 | 1.000 | 0.500 | 0.330 | 1.000 | 1.000 | 0.411 |
| 35 | epidermis-hair-uHF_joost | 1.000 | 1.000 | 1.000 | 1.000 | 0.368 | 0.322 | 1.000 | 1.000 | 1.000 |
| 36 | planaria-epidermis-differentiation_plass | 1.000 |  | 0.560 | 1.000 | 0.281 | 0.254 | 0.560 |  |  |
| 37 | cortical-interneuron-differentiation_frazer | 0.654 | 0.710 | 0.921 | 0.555 | 0.518 | 0.389 | 0.502 | 0.914 | 0.693 |
| 38 | thymus-t-cell-differentiation_mca | 1.000 | 0.593 | 0.560 | 1.000 | 0.464 | 0.310 | 0.560 | 1.000 | 0.451 |
| 39 | planaria-muscle-differentiation_plass | 1.000 | 1.000 | 1.000 | 1.000 | 0.208 | 0.322 | 1.000 | 1.000 | 1.000 |
| 40 | hematopoiesis-clusters_olsson | 0.908 | 0.340 | 0.340 | 0.340 | 0.634 | 0.446 | 0.524 | 0.340 | 0.872 |
| 41 | planaria-pair-8_plass | 0.541 |  | 0.906 | 0.541 | 0.550 | 0.506 | 0.541 | 0.541 |  |
| 42 | hepatoblast-differentiation_yang | 1.000 | 0.870 | 0.609 | 0.616 | 0.544 | 0.318 | 0.665 | 0.779 | 0.616 |
| 43 | distal-lung-epithelium_treutlein | 1.000 |  | 1.000 |  | 1.000 | 0.322 | 1.000 | 1.000 | 1.000 |
| 44 | bone-marrow-mesenchyme-erythrocyte-differentiation_mca | 1.000 | 1.000 | 1.000 | 1.000 | 0.650 | 0.322 | 1.000 | 1.000 | 0.581 |
| 45 | planaria-phagocyte-differentiation_plass | 1.000 |  | 1.000 | 1.000 | 0.556 | 1.000 | 1.000 |  | 0.552 |
| 46 | germline-human-male_li | 1.000 | 1.000 | 1.000 | 1.000 | 0.536 | 1.000 | 1.000 | 1.000 | 0.419 |
| 47 | dentate-gyrus-neurogenesis_hochgerner | 1.000 |  | 1.000 | 1.000 | 0.355 | 0.347 | 0.560 |  | 1.000 |
| 48 | planaria-pair-10_plass | 0.903 |  | 0.864 | 0.325 | 0.705 | 0.602 | 0.860 |  |  |
| 49 | planaria-pair-9_plass | 0.825 |  | 0.667 | 0.244 | 0.827 | 0.649 | 0.777 | 0.623 |  |
| 50 | planaria-pair-11_plass | 0.916 |  | 0.545 | 0.365 | 0.633 | 0.547 | 0.836 |  |  |
| 51 | neonatal-inner-ear-TEC-HSC_burns | 1.000 | 1.000 | 1.000 | 1.000 | 1.000 | 0.322 | 1.000 | 1.000 | 0.390 |
| 52 | planaria-neuron-differentiation_plass | 1.000 | 0.205 | 0.205 | 0.205 | 0.697 | 0.554 | 0.450 | 0.478 | 0.205 |
| 53 | placenta-trophoblast-differentiation_mca | 1.000 | 0.565 | 0.565 | 0.565 | 0.522 | 0.580 | 0.565 | 0.565 | 0.565 |
| 54 | planaria-parenchyme-differentiation_plass | 1.000 | 0.454 | 0.718 | 0.363 | 0.597 | 0.595 | 0.718 | 0.691 | 0.880 |
|  | Average | **0.922** | 0.827 | 0.829 | 0.779 | 0.593 | 0.472 | 0.822 | 0.811 | 0.635 |

**Table 3. F1 branches of real datasets**

|  |  | F1 branches | | | | | | | | |
| --- | --- | --- | --- | --- | --- | --- | --- | --- | --- | --- |
|  | dataset_name | scCRT | scTite | Slingshot | scShaper | Monocle3 | PAGA | TSCAN | scTEP | Totem |
| 1 | aging-hsc-young_kowalczyk | 1.000 | 1.000 | 1.000 | 1.000 | 1.000 | 0.370 | 1.000 | 1.000 | 0.655 |
| 2 | NKT-differentiation_engel | 0.670 | 0.387 | 0.458 | 0.387 | 0.559 | 0.440 | 0.387 | 0.551 | 0.328 |
| 3 | pancreatic-beta-cell-maturation_zhang | 1.000 | 0.423 | 1.000 | 1.000 | 0.131 | 0.100 | 1.000 | 1.000 | 0.410 |
| 4 | stimulated-dendritic-cells-PIC_shalek | 0.428 | 0.400 | 0.404 | 1.000 | 0.159 | 0.126 | 0.374 | 0.399 | 0.320 |
| 5 | myoblast-differentiation_trapnell | 1.000 | 1.000 | 1.000 | 1.000 | 0.404 | 0.212 | 1.000 | 0.391 | 0.398 |
| 6 | hematopoiesis-gates_olsson | 1.000 | 1.000 | 1.000 | 1.000 | 0.426 | 0.349 | 1.000 | 1.000 | 0.202 |
| 7 | developing-dendritic-cells_schlitzer | 1.000 | 1.000 | 1.000 | 1.000 | 0.234 | 0.367 | 1.000 | 1.000 | 1.000 |
| 8 | mESC-differentiation_hayashi | 1.000 | 0.413 | 1.000 | 1.000 | 1.000 | 1.000 | 1.000 | 1.000 | 0.376 |
| 9 | psc-astrocyte-maturation-neuron_sloan | 1.000 | 0.639 | 1.000 | 1.000 | 0.200 | 0.231 | 1.000 | 0.605 | 0.295 |
| 10 | germline-human-both_guo | 0.454 | 0.429 | 0.317 | 0.453 | 0.179 | 0.179 | 0.447 | 0.350 | 0.294 |
| 11 | macrophage-salmonella_saliba | 0.723 | 0.405 | 0.424 |  | 0.405 | 0.270 | 0.493 | 0.405 | 0.405 |
| 12 | psc-astrocyte-maturation-glia_sloan | 0.639 | 0.571 | 0.365 | 1.000 | 0.286 | 0.266 | 0.382 | 0.368 | 0.161 |
| 13 | aging-hsc-old_kowalczyk | 1.000 | 1.000 | 1.000 | 1.000 | 0.459 | 0.359 | 1.000 | 1.000 | 1.000 |
| 14 | stimulated-dendritic-cells-LPS_shalek | 0.555 | 1.000 | 1.000 | 1.000 | 0.114 | 0.153 | 0.396 | 0.515 | 1.000 |
| 15 | pancreatic-alpha-cell-maturation_zhang | 1.000 | 1.000 | 1.000 | 1.000 | 0.401 | 0.108 | 1.000 | 0.392 | 0.463 |
| 16 | stimulated-dendritic-cells-PAM_shalek | 1.000 | 0.515 | 0.524 | 1.000 | 0.170 | 0.153 | 0.546 | 0.529 | 0.368 |
| 17 | human-embryos_petropoulos | 1.000 | 1.000 | 1.000 | 1.000 | 0.149 | 1.000 | 1.000 |  | 0.206 |
| 18 | trophectoderm-monkey_nakamura | 1.000 | 0.594 | 1.000 | 1.000 | 0.425 | 1.000 | 1.000 | 1.000 | 0.262 |
| 19 | planaria-pair-4_plass | 0.331 |  | 0.288 | 0.217 | 0.103 | 0.209 | 0.370 | 0.179 |  |
| 20 | fibroblast-reprogramming_treutlein | 0.586 | 0.604 | 0.874 | 0.447 | 0.321 | 0.305 | 0.893 | 0.588 | 0.672 |
| 21 | planaria-pharynx-differentiation_plass | 1.000 |  | 1.000 | 1.000 | 0.413 | 1.000 | 1.000 |  | 0.181 |
| 22 | embronic-mesenchyme-neuron-differentiation_mca | 0.566 | 0.353 | 0.286 | 0.286 | 0.435 | 0.290 | 0.348 | 0.380 | 0.401 |
| 23 | planaria-pair-2_plass | 0.415 |  | 0.250 | 0.148 | 0.153 | 0.277 | 0.464 | 0.203 |  |
| 24 | epidermis-hair-IFE_joost | 1.000 | 1.000 | 1.000 | 1.000 | 0.222 | 0.219 | 1.000 | 1.000 | 0.371 |
| 25 | planaria-pair-1_plass | 0.366 |  | 0.263 | 0.660 | 0.094 | 0.137 | 0.411 | 0.195 |  |
| 26 | olfactory-projection-neurons-DA1_horns | 1.000 | 0.513 | 1.000 | 1.000 | 0.429 | 0.301 | 0.355 | 1.000 | 0.344 |
| 27 | oligodendrocyte-differentiation-clusters_marques | 0.364 |  | 0.618 | 1.000 | 0.205 | 0.479 | 1.000 | 1.000 | 1.000 |
| 28 | neonatal-inner-ear-TEC-SC_burns | 1.000 | 1.000 | 1.000 | 1.000 | 0.429 | 0.354 | 1.000 | 1.000 | 0.597 |
| 29 | planaria-pair-3_plass | 0.553 |  | 0.463 | 0.277 | 0.212 | 0.456 | 0.675 | 0.265 |  |
| 30 | neonatal-inner-ear-SC-HC_burns | 1.000 | 1.000 | 1.000 | 1.000 | 1.000 | 0.324 | 1.000 | 1.000 | 0.221 |
| 31 | planaria-pair-14_plass | 0.361 |  | 0.263 | 0.215 | 0.116 | 0.192 | 0.472 | 0.172 |  |
| 32 | oligodendrocyte-differentiation-subclusters_marques | 0.455 |  | 0.370 | 0.216 | 0.150 | 0.103 | 0.286 | 0.259 |  |
| 33 | germline-human-female_li | 1.000 |  | 1.000 | 1.000 | 0.220 | 1.000 | 1.000 | 1.000 | 0.219 |
| 34 | epidermis-hair-spatial_joost | 1.000 | 0.580 | 1.000 | 1.000 | 0.191 | 0.214 | 1.000 | 1.000 | 0.322 |
| 35 | epidermis-hair-uHF_joost | 0.580 | 1.000 | 0.580 | 1.000 | 0.143 | 0.389 | 1.000 | 1.000 | 0.575 |
| 36 | planaria-epidermis-differentiation_plass | 1.000 |  | 0.365 | 1.000 | 0.084 | 0.230 | 0.472 |  |  |
| 37 | cortical-interneuron-differentiation_frazer | 0.407 | 0.297 | 0.465 | 0.438 | 0.234 | 0.162 | 0.397 | 0.314 | 0.410 |
| 38 | thymus-t-cell-differentiation_mca | 0.416 | 0.639 | 0.599 | 0.575 | 0.361 | 0.272 | 0.373 | 0.575 | 0.337 |
| 39 | planaria-muscle-differentiation_plass | 0.639 | 0.639 | 0.639 | 0.639 | 0.130 | 0.365 | 0.639 | 0.639 | 0.403 |
| 40 | hematopoiesis-clusters_olsson | 0.432 | 0.324 | 0.347 | 0.234 | 0.236 | 0.198 | 0.429 | 0.326 | 0.430 |
| 41 | planaria-pair-8_plass | 0.338 |  | 0.430 | 0.376 | 0.230 | 0.349 | 0.409 | 0.280 |  |
| 42 | hepatoblast-differentiation_yang | 0.676 | 0.502 | 0.485 | 0.388 | 0.242 | 0.129 | 0.524 | 0.380 | 0.388 |
| 43 | distal-lung-epithelium_treutlein | 0.940 |  | 0.845 |  | 0.629 | 0.499 | 0.759 | 0.771 | 0.556 |
| 44 | bone-marrow-mesenchyme-erythrocyte-differentiation_mca | 1.000 | 1.000 | 1.000 | 1.000 | 0.178 | 0.428 | 1.000 | 1.000 | 0.382 |
| 45 | planaria-phagocyte-differentiation_plass | 1.000 |  | 1.000 | 1.000 | 0.391 | 1.000 | 1.000 |  | 0.351 |
| 46 | germline-human-male_li | 1.000 | 1.000 | 1.000 | 1.000 | 0.236 | 1.000 | 1.000 | 1.000 | 0.202 |
| 47 | dentate-gyrus-neurogenesis_hochgerner | 1.000 |  | 1.000 | 1.000 | 0.142 | 0.269 | 0.476 |  | 1.000 |
| 48 | planaria-pair-10_plass | 0.341 |  | 0.242 | 0.192 | 0.191 | 0.245 | 0.417 |  |  |
| 49 | planaria-pair-9_plass | 0.337 |  | 0.225 | 0.137 | 0.223 | 0.249 | 0.447 | 0.211 |  |
| 50 | planaria-pair-11_plass | 0.538 |  | 0.284 | 0.242 | 0.240 | 0.228 | 0.499 |  |  |
| 51 | neonatal-inner-ear-TEC-HSC_burns | 1.000 | 1.000 | 1.000 | 1.000 | 1.000 | 0.359 | 1.000 | 1.000 | 0.287 |
| 52 | planaria-neuron-differentiation_plass | 0.553 | 0.229 | 0.265 | 0.265 | 0.182 | 0.266 | 0.390 | 0.205 | 0.265 |
| 53 | placenta-trophoblast-differentiation_mca | 0.503 | 0.456 | 0.577 | 0.456 | 0.290 | 0.375 | 0.480 | 0.553 | 0.413 |
| 54 | planaria-parenchyme-differentiation_plass | 0.579 | 0.294 | 0.482 | 0.335 | 0.303 | 0.312 | 0.491 | 0.451 | 0.544 |
|  | Average | **0.736** | 0.681 | 0.685 | 0.723 | 0.316 | 0.368 | 0.694 | 0.627 | 0.442 |

**Table 4. F1 milestones of real datasets**

|  |  | F1 milestones | | | | | | | | |
| --- | --- | --- | --- | --- | --- | --- | --- | --- | --- | --- |
|  | dataset_name | scCRT | scTite | Slingshot | scShaper | Monocle3 | PAGA | TSCAN | scTEP | Totem |
| 1 | aging-hsc-young_kowalczyk | 0.782 | 0.634 | 0.728 | 0.721 | 0.687 | 0.644 | 0.781 | 0.480 | 0.719 |
| 2 | NKT-differentiation_engel | 0.842 | 0.430 | 0.792 | 0.484 | 0.440 | 0.864 | 0.460 | 0.653 | 0.606 |
| 3 | pancreatic-beta-cell-maturation_zhang | 0.428 | 0.472 | 0.858 | 0.506 | 0.210 | 0.299 | 0.819 | 0.524 | 0.413 |
| 4 | stimulated-dendritic-cells-PIC_shalek | 0.491 | 0.395 | 0.474 | 0.771 | 0.258 | 0.426 | 0.468 | 0.446 | 0.389 |
| 5 | myoblast-differentiation_trapnell | 0.823 | 0.555 | 0.414 | 0.394 | 0.387 | 0.455 | 0.676 | 0.388 | 0.300 |
| 6 | hematopoiesis-gates_olsson | 0.775 | 0.663 | 0.721 | 0.784 | 0.455 | 0.576 | 0.789 | 0.731 | 0.282 |
| 7 | developing-dendritic-cells_schlitzer | 0.699 | 0.705 | 0.730 | 0.834 | 0.342 | 0.633 | 0.693 | 0.711 | 0.869 |
| 8 | mESC-differentiation_hayashi | 0.863 | 0.458 | 0.819 | 0.822 | 0.659 | 0.776 | 0.815 | 0.990 | 0.444 |
| 9 | psc-astrocyte-maturation-neuron_sloan | 0.538 | 0.345 | 0.532 | 0.376 | 0.172 | 0.492 | 0.473 | 0.307 | 0.220 |
| 10 | germline-human-both_guo | 0.601 | 0.466 | 0.362 | 0.433 | 0.348 | 0.553 | 0.448 | 0.355 | 0.406 |
| 11 | macrophage-salmonella_saliba | 0.967 | 0.422 | 0.613 |  | 0.575 | 0.705 | 0.607 | 0.434 | 0.425 |
| 12 | psc-astrocyte-maturation-glia_sloan | 0.468 | 0.447 | 0.335 | 0.560 | 0.259 | 0.542 | 0.328 | 0.405 | 0.294 |
| 13 | aging-hsc-old_kowalczyk | 0.685 | 0.575 | 0.605 | 0.593 | 0.439 | 0.614 | 0.648 | 0.589 | 0.690 |
| 14 | stimulated-dendritic-cells-LPS_shalek | 0.408 | 0.635 | 0.888 | 0.967 | 0.182 | 0.447 | 0.416 | 0.591 | 0.427 |
| 15 | pancreatic-alpha-cell-maturation_zhang | 0.594 | 0.441 | 0.830 | 0.454 | 0.361 | 0.362 | 0.814 | 0.269 | 0.280 |
| 16 | stimulated-dendritic-cells-PAM_shalek | 0.824 | 0.547 | 0.551 | 0.559 | 0.229 | 0.375 | 0.570 | 0.488 | 0.415 |
| 17 | human-embryos_petropoulos | 0.851 | 0.825 | 0.789 | 0.854 | 0.220 | 0.857 | 0.773 |  | 0.251 |
| 18 | trophectoderm-monkey_nakamura | 0.668 | 0.747 | 0.600 | 0.398 | 0.585 | 0.650 | 0.585 | 0.494 | 0.451 |
| 19 | planaria-pair-4_plass | 0.635 |  | 0.267 | 0.152 | 0.149 | 0.636 | 0.409 | 0.263 |  |
| 20 | fibroblast-reprogramming_treutlein | 0.564 | 0.644 | 0.819 | 0.458 | 0.427 | 0.610 | 0.760 | 0.696 | 0.706 |
| 21 | planaria-pharynx-differentiation_plass | 0.934 |  | 0.950 | 0.758 | 0.573 | 0.873 | 0.802 |  | 0.288 |
| 22 | embronic-mesenchyme-neuron-differentiation_mca | 0.595 | 0.332 | 0.352 | 0.273 | 0.403 | 0.625 | 0.389 | 0.324 | 0.418 |
| 23 | planaria-pair-2_plass | 0.637 |  | 0.276 | 0.133 | 0.217 | 0.645 | 0.458 | 0.257 |  |
| 24 | epidermis-hair-IFE_joost | 0.868 | 0.449 | 0.763 | 0.461 | 0.243 | 0.465 | 0.669 | 0.451 | 0.337 |
| 25 | planaria-pair-1_plass | 0.635 |  | 0.398 | 0.361 | 0.134 | 0.440 | 0.445 | 0.372 |  |
| 26 | olfactory-projection-neurons-DA1_horns | 0.862 | 0.594 | 0.806 | 0.707 | 0.573 | 0.418 | 0.455 | 0.607 | 0.522 |
| 27 | oligodendrocyte-differentiation-clusters_marques | 0.560 |  | 0.650 | 0.490 | 0.212 | 0.694 | 0.923 | 0.642 | 0.646 |
| 28 | neonatal-inner-ear-TEC-SC_burns | 0.889 | 0.808 | 0.848 | 0.751 | 0.328 | 0.555 | 0.889 | 0.848 | 0.668 |
| 29 | planaria-pair-3_plass | 0.661 |  | 0.474 | 0.195 | 0.333 | 0.765 | 0.675 | 0.397 |  |
| 30 | neonatal-inner-ear-SC-HC_burns | 1.000 | 0.983 | 1.000 | 0.983 | 0.983 | 0.566 | 0.966 | 0.983 | 0.399 |
| 31 | planaria-pair-14_plass | 0.511 |  | 0.324 | 0.177 | 0.172 | 0.577 | 0.508 | 0.237 |  |
| 32 | oligodendrocyte-differentiation-subclusters_marques | 0.414 |  | 0.392 | 0.205 | 0.182 | 0.311 | 0.362 | 0.238 |  |
| 33 | germline-human-female_li | 0.863 |  | 0.826 | 0.637 | 0.173 | 0.874 | 0.814 | 0.644 | 0.366 |
| 34 | epidermis-hair-spatial_joost | 0.378 | 0.417 | 0.678 | 0.422 | 0.253 | 0.529 | 0.708 | 0.659 | 0.283 |
| 35 | epidermis-hair-uHF_joost | 0.564 | 0.423 | 0.501 | 0.401 | 0.237 | 0.565 | 0.495 | 0.441 | 0.544 |
| 36 | planaria-epidermis-differentiation_plass | 0.827 |  | 0.421 | 0.577 | 0.154 | 0.463 | 0.479 |  |  |
| 37 | cortical-interneuron-differentiation_frazer | 0.532 | 0.354 | 0.494 | 0.391 | 0.317 | 0.473 | 0.545 | 0.401 | 0.547 |
| 38 | thymus-t-cell-differentiation_mca | 0.839 | 0.605 | 0.531 | 0.546 | 0.318 | 0.643 | 0.466 | 0.540 | 0.517 |
| 39 | planaria-muscle-differentiation_plass | 0.458 | 0.583 | 0.530 | 0.578 | 0.225 | 0.670 | 0.477 | 0.568 | 0.356 |
| 40 | hematopoiesis-clusters_olsson | 0.465 | 0.286 | 0.329 | 0.354 | 0.239 | 0.564 | 0.398 | 0.372 | 0.352 |
| 41 | planaria-pair-8_plass | 0.503 |  | 0.548 | 0.233 | 0.278 | 0.564 | 0.577 | 0.305 |  |
| 42 | hepatoblast-differentiation_yang | 0.507 | 0.428 | 0.483 | 0.373 | 0.341 | 0.346 | 0.523 | 0.470 | 0.427 |
| 43 | distal-lung-epithelium_treutlein | 0.877 |  | 0.846 |  | 0.492 | 0.903 | 0.757 | 0.748 | 0.508 |
| 44 | bone-marrow-mesenchyme-erythrocyte-differentiation_mca | 0.651 | 0.468 | 0.698 | 0.413 | 0.252 | 0.612 | 0.388 | 0.402 | 0.473 |
| 45 | planaria-phagocyte-differentiation_plass | 0.880 |  | 0.855 | 0.730 | 0.525 | 0.909 | 0.792 |  | 0.446 |
| 46 | germline-human-male_li | 0.725 | 0.948 | 0.732 | 0.945 | 0.406 | 0.783 | 0.702 | 0.951 | 0.348 |
| 47 | dentate-gyrus-neurogenesis_hochgerner | 0.855 |  | 0.789 | 0.615 | 0.214 | 0.453 | 0.430 |  | 0.839 |
| 48 | planaria-pair-10_plass | 0.600 |  | 0.357 | 0.184 | 0.270 | 0.549 | 0.513 |  |  |
| 49 | planaria-pair-9_plass | 0.657 |  | 0.226 | 0.135 | 0.325 | 0.647 | 0.482 | 0.285 |  |
| 50 | planaria-pair-11_plass | 0.746 |  | 0.387 | 0.186 | 0.305 | 0.580 | 0.515 |  |  |
| 51 | neonatal-inner-ear-TEC-HSC_burns | 0.888 | 0.888 | 0.867 | 0.727 | 0.867 | 0.663 | 0.931 | 0.910 | 0.418 |
| 52 | planaria-neuron-differentiation_plass | 0.757 | 0.292 | 0.319 | 0.327 | 0.237 | 0.697 | 0.475 | 0.385 | 0.319 |
| 53 | placenta-trophoblast-differentiation_mca | 0.750 | 0.471 | 0.416 | 0.479 | 0.359 | 0.712 | 0.431 | 0.485 | 0.306 |
| 54 | planaria-parenchyme-differentiation_plass | 0.812 | 0.514 | 0.736 | 0.375 | 0.443 | 0.764 | 0.758 | 0.625 | 0.644 |
|  | Average | **0.689** | 0.547 | 0.602 | 0.505 | 0.351 | 0.600 | 0.602 | 0.518 | 0.455 |

**Table 5. Details of synthetic datasets**

|  | types | dataset_name | cell_nums | trajectory_type |
| --- | --- | --- | --- | --- |
| 1 | synthetic-dyntoy | multifurcating_8 | 7,971 | tree |
| 2 | synthetic-dyntoy | bifurcating_4 | 2,011 | bifurcation |
| 3 | synthetic-dyntoy | multifurcating_7 | 562 | tree |
| 4 | synthetic-dyntoy | bifurcating_8 | 7,674 | bifurcation |
| 5 | synthetic-dyntoy | multifurcating_10 | 550 | tree |
| 6 | synthetic-dyntoy | tree_5 | 2,360 | tree |
| 7 | synthetic-dyntoy | multifurcating_4 | 2,064 | multifurcation |
| 8 | synthetic-dyntoy | multifurcating_9 | 4,861 | tree |
| 9 | synthetic-dyntoy | binary_tree_9 | 5,018 | tree |
| 10 | synthetic-dyntoy | multifurcating_5 | 2,399 | bifurcation |
| 11 | synthetic-dyntoy | tree_3 | 362 | tree |
| 12 | synthetic-dyntoy | linear_8 | 8,282 | linear |
| 13 | synthetic-dyntoy | linear_2 | 249 | linear |
| 14 | synthetic-dyntoy | linear_5 | 2,405 | linear |
| 15 | synthetic-dyntoy | multifurcating_3 | 362 | tree |
| 16 | synthetic-dyntoy | linear_10 | 555 | linear |
| 17 | synthetic-dyntoy | binary_tree_2 | 250 | tree |
| 18 | synthetic-dyntoy | binary_tree_4 | 2,057 | tree |
| 19 | synthetic-dyntoy | bifurcating_6 | 4,925 | bifurcation |
| 20 | synthetic-dyntoy | tree_10 | 550 | tree |
| 21 | synthetic-dyntoy | binary_tree_7 | 575 | tree |
| 22 | synthetic-dyntoy | bifurcating_3 | 359 | bifurcation |
| 23 | synthetic-dyntoy | linear_7 | 575 | linear |
| 24 | synthetic-dyntoy | binary_tree_8 | 8,249 | tree |
| 25 | synthetic-dyntoy | tree_6 | 4,880 | tree |
| 26 | synthetic-dyntoy | binary_tree_5 | 2,378 | tree |
| 27 | synthetic-dyntoy | tree_2 | 251 | tree |
| 28 | synthetic-dyntoy | linear_1 | 927 | linear |
| 29 | synthetic-dyntoy | bifurcating_2 | 247 | bifurcation |
| 30 | synthetic-dyntoy | bifurcating_10 | 541 | bifurcation |
| 31 | synthetic-dyntoy | multifurcating_6 | 4,945 | tree |
| 32 | synthetic-dyntoy | linear_3 | 359 | linear |
| 33 | synthetic-dyntoy | tree_4 | 2,062 | tree |
| 34 | synthetic-dyntoy | diverging_converging_1 | 930 | tree |
| 35 | synthetic-dyntoy | tree_8 | 8,246 | tree |
| 36 | synthetic-dyntoy | tree_7 | 558 | tree |
| 37 | synthetic-dyntoy | tree_1 | 905 | tree |
| 38 | synthetic-dyntoy | bifurcating_1 | 926 | bifurcation |
| 39 | synthetic-dyntoy | diverging_converging_9 | 4,739 | tree |
| 40 | synthetic-dyntoy | bifurcating_5 | 2,197 | bifurcation |
| 41 | synthetic-dyntoy | multifurcating_1 | 879 | tree |
| 42 | synthetic-dyntoy | linear_4 | 1,950 | linear |
| 43 | synthetic-dyntoy | binary_tree_10 | 550 | tree |
| 44 | synthetic-dyntoy | bifurcating_7 | 575 | bifurcation |
| 45 | synthetic-dyntoy | binary_tree_1 | 929 | tree |
| 46 | synthetic-dyntoy | binary_tree_6 | 4,865 | tree |
| 47 | synthetic-dyntoy | bifurcating_9 | 4,893 | bifurcation |
| 48 | synthetic-dyntoy | tree_9 | 4,963 | tree |
| 49 | synthetic-dyntoy | multifurcating_2 | 247 | tree |
| 50 | synthetic-dyntoy | linear_9 | 4,923 | linear |
| 51 | synthetic-dyntoy | linear_6 | 4,956 | linear |
| 52 | synthetic-prosstt | tree_3 | 2,560 | tree |
| 53 | synthetic-prosstt | binary_tree_3 | 2,597 | tree |
| 54 | synthetic-prosstt | multifurcating_3 | 2,590 | tree |
| 55 | synthetic-prosstt | bifurcating_6 | 972 | bifurcation |
| 56 | synthetic-prosstt | tree_6 | 973 | tree |
| 57 | synthetic-prosstt | linear_1 | 488 | linear |
| 58 | synthetic-prosstt | multifurcating_6 | 981 | multifurcation |
| 59 | synthetic-prosstt | linear_3 | 2,593 | linear |
| 60 | synthetic-prosstt | tree_1 | 490 | tree |
| 61 | synthetic-prosstt | bifurcating_1 | 486 | bifurcation |
| 62 | synthetic-prosstt | binary_tree_1 | 475 | tree |
| 63 | synthetic-prosstt | binary_tree_6 | 982 | tree |
| 64 | synthetic-prosstt | linear_6 | 976 | linear |
| 65 | synthetic-splatter | bifurcating_3_2 | 359 | bifurcation |
| 66 | synthetic-splatter | bifurcating_5_2 | 2,425 | bifurcation |
| 67 | synthetic-splatter | linear_5 | 2,428 | linear |
| 68 | synthetic-splatter | linear_10 | 558 | linear |
| 69 | synthetic-splatter | binary_tree_4 | 2,075 | tree |
| 70 | synthetic-splatter | linear_7 | 571 | linear |
| 71 | synthetic-splatter | linear_1 | 918 | linear |
| 72 | synthetic-splatter | binary_tree_3_2 | 359 | tree |
| 73 | synthetic-splatter | bifurcating_10 | 555 | bifurcation |
| 74 | synthetic-splatter | linear_3_2 | 359 | linear |
| 75 | synthetic-splatter | bifurcating_1_2 | 922 | bifurcation |
| 76 | synthetic-splatter | multifurcating_1 | 920 | tree |
| 77 | synthetic-splatter | linear_4 | 2,073 | linear |
| 78 | synthetic-splatter | bifurcating_4_2 | 2,063 | bifurcation |
| 79 | synthetic-splatter | bifurcating_2_2 | 244 | bifurcation |
| 80 | synthetic-splatter | bifurcating_7_2 | 573 | bifurcation |
| 81 | synthetic-splatter | multifurcating_3_2 | 359 | tree |

**Table 6. HIM of synthetic datasets**

|  |  | HIM | | | | | | | | |
| --- | --- | --- | --- | --- | --- | --- | --- | --- | --- | --- |
|  | dataset_name | scCRT | scTite | Slingshot | scShaper | Monocle3 | PAGA | TSCAN | scTEP | Totem |
| 1 | multifurcating_8 | 1.000 |  | 0.258 | 0.376 | 0.641 | 0.594 | 0.744 |  |  |
| 2 | bifurcating_4 | 1.000 | 0.555 | 1.000 | 0.555 | 0.844 | 0.555 | 1.000 | 0.848 | 0.849 |
| 3 | multifurcating_7 | 0.973 | 0.525 | 0.708 | 0.353 | 0.769 | 0.799 | 0.293 | 0.756 | 0.826 |
| 4 | bifurcating_8 | 1.000 |  | 0.555 | 0.616 | 0.461 | 0.555 | 0.555 |  |  |
| 5 | multifurcating_10 | 1.000 | 0.466 | 1.000 | 0.466 | 0.769 | 0.650 | 0.533 | 0.466 | 0.908 |
| 6 | tree_5 | 1.000 | 0.615 | 0.319 | 0.463 | 0.674 | 0.652 | 0.511 | 0.609 | 0.463 |
| 7 | multifurcating_4 | 0.723 | 0.596 | 0.363 | 0.596 | 0.635 | 0.421 | 0.363 | 0.596 | 0.636 |
| 8 | multifurcating_9 | 1.000 |  | 0.421 | 0.423 | 0.632 | 0.629 | 0.421 |  |  |
| 9 | binary_tree_9 | 1.000 |  | 1.000 | 0.363 | 0.698 | 0.851 | 0.841 |  |  |
| 10 | multifurcating_5 | 1.000 | 0.906 | 1.000 | 0.560 | 0.675 | 1.000 | 1.000 | 0.866 | 0.915 |
| 11 | tree_3 | 0.863 | 0.695 | 0.851 | 0.352 | 0.914 | 0.787 | 0.421 | 0.708 | 0.890 |
| 12 | linear_8 | 1.000 |  | 1.000 | 1.000 | 0.795 |  | 1.000 |  |  |
| 13 | linear_2 | 1.000 | 1.000 | 1.000 | 1.000 | 0.469 | 1.000 | 1.000 | 1.000 | 1.000 |
| 14 | linear_5 | 1.000 | 1.000 | 1.000 | 1.000 | 0.488 | 1.000 | 1.000 | 1.000 | 1.000 |
| 15 | multifurcating_3 | 0.784 | 0.636 | 0.891 | 0.357 | 0.862 | 0.767 | 0.494 | 0.776 | 0.879 |
| 16 | linear_10 | 1.000 | 1.000 | 1.000 | 1.000 | 0.672 | 0.420 | 1.000 | 1.000 | 1.000 |
| 17 | binary_tree_2 | 1.000 |  | 1.000 | 0.320 | 0.727 | 0.753 | 0.709 | 0.461 | 0.887 |
| 18 | binary_tree_4 | 1.000 | 0.356 | 0.709 | 0.356 | 0.726 | 0.676 | 0.548 | 0.690 | 0.356 |
| 19 | bifurcating_6 | 1.000 |  | 1.000 | 0.565 | 0.667 | 1.000 | 1.000 |  |  |
| 20 | tree_10 | 0.920 | 0.562 | 0.737 | 0.331 | 0.792 | 0.677 | 0.622 | 0.667 | 0.672 |
| 21 | binary_tree_7 | 1.000 | 0.838 | 1.000 | 0.342 | 0.829 | 0.831 | 0.581 | 0.579 | 0.795 |
| 22 | bifurcating_3 | 1.000 | 0.561 | 1.000 | 0.561 | 0.621 | 1.000 | 1.000 | 0.561 | 0.598 |
| 23 | linear_7 | 1.000 | 1.000 | 1.000 | 1.000 | 0.588 | 1.000 | 1.000 | 1.000 | 1.000 |
| 24 | binary_tree_8 | 1.000 | 0.434 | 0.763 | 0.434 | 0.652 | 0.777 | 0.483 |  |  |
| 25 | tree_6 | 0.933 |  | 0.939 | 0.321 | 0.836 | 0.805 | 0.609 |  |  |
| 26 | binary_tree_5 | 1.000 | 0.389 | 0.361 | 0.389 | 0.804 | 0.701 | 0.386 | 0.807 | 0.389 |
| 27 | tree_2 | 0.804 | 0.466 | 0.818 | 0.343 | 0.452 | 0.684 | 0.850 | 0.465 | 0.860 |
| 28 | linear_1 | 1.000 | 1.000 | 1.000 | 1.000 | 0.393 | 1.000 | 1.000 | 1.000 | 1.000 |
| 29 | bifurcating_2 | 1.000 | 0.581 | 1.000 | 0.885 | 0.525 | 0.272 | 0.555 | 0.885 | 0.548 |
| 30 | bifurcating_10 | 1.000 | 0.839 | 1.000 | 0.529 | 0.764 | 0.555 | 0.555 | 0.743 | 0.794 |
| 31 | multifurcating_6 | 1.000 |  | 0.919 | 0.397 | 0.806 | 0.778 | 0.673 |  |  |
| 32 | linear_3 | 1.000 | 1.000 | 1.000 | 1.000 | 0.722 | 0.494 | 1.000 | 1.000 | 1.000 |
| 33 | tree_4 | 1.000 | 0.417 | 1.000 | 0.417 | 0.648 | 0.862 | 0.483 | 0.618 | 0.829 |
| 34 | diverging_converging_1 | 1.000 | 0.578 | 1.000 | 0.462 | 0.732 | 0.777 | 0.483 | 0.576 | 0.839 |
| 35 | tree_8 | 0.825 |  | 0.663 | 0.302 | 0.794 | 0.714 | 0.662 |  |  |
| 36 | tree_7 | 1.000 |  | 0.783 | 0.405 | 0.785 | 0.498 | 0.603 | 0.858 | 0.405 |
| 37 | tree_1 | 1.000 | 0.552 | 0.747 | 0.394 | 0.696 | 0.645 | 0.319 | 0.793 | 0.394 |
| 38 | bifurcating_1 | 1.000 | 0.592 | 1.000 | 0.592 | 0.621 | 0.555 | 1.000 | 0.592 | 0.896 |
| 39 | diverging_converging_9 | 0.883 |  | 0.278 | 0.330 | 0.585 | 0.571 | 0.474 |  |  |
| 40 | bifurcating_5 | 1.000 | 0.982 | 0.555 | 0.563 | 0.575 | 0.272 | 1.000 | 0.982 | 0.707 |
| 41 | multifurcating_1 | 0.853 |  | 0.533 | 0.439 | 0.764 | 0.410 | 0.532 | 0.596 | 0.439 |
| 42 | linear_4 | 1.000 | 1.000 | 1.000 | 1.000 | 0.562 | 0.330 | 1.000 | 1.000 | 1.000 |
| 43 | binary_tree_10 | 1.000 | 0.676 | 1.000 | 0.346 | 0.788 | 0.783 | 0.713 | 0.664 | 0.661 |
| 44 | bifurcating_7 | 1.000 | 0.683 | 1.000 | 0.683 | 0.829 | 1.000 | 1.000 | 0.683 | 0.712 |
| 45 | binary_tree_1 | 1.000 | 0.328 | 0.859 | 0.328 | 0.835 | 0.754 | 0.724 | 0.472 | 0.654 |
| 46 | binary_tree_6 | 1.000 |  | 0.868 | 0.311 | 0.740 | 0.767 | 0.413 |  |  |
| 47 | bifurcating_9 | 1.000 |  | 1.000 | 0.569 | 0.441 | 0.555 | 1.000 |  |  |
| 48 | tree_9 | 1.000 |  | 0.667 | 0.376 | 0.813 | 0.703 | 0.436 |  |  |
| 49 | multifurcating_2 | 0.900 |  | 0.762 | 0.286 | 0.343 | 0.548 | 0.656 | 0.691 | 0.286 |
| 50 | linear_9 | 1.000 |  | 1.000 | 1.000 | 0.439 | 0.204 | 1.000 |  |  |
| 51 | linear_6 | 1.000 |  | 1.000 | 1.000 | 1.000 | 0.463 | 1.000 |  |  |
| 52 | tree_3 | 0.918 | 0.697 | 0.885 | 0.366 | 0.893 | 0.790 | 0.630 | 0.689 | 0.742 |
| 53 | binary_tree_3 | 1.000 | 0.829 | 0.885 | 0.431 | 0.840 | 0.773 | 0.658 | 0.910 | 0.844 |
| 54 | multifurcating_3 | 1.000 | 0.322 | 0.897 | 0.322 | 0.831 | 0.929 | 0.770 |  | 0.794 |
| 55 | bifurcating_6 | 1.000 | 0.905 | 1.000 | 0.597 | 0.845 | 1.000 | 0.555 | 0.984 | 0.863 |
| 56 | tree_6 | 1.000 | 0.654 | 0.902 | 0.343 | 0.781 | 0.838 | 0.819 |  | 0.604 |
| 57 | linear_1 | 1.000 | 1.000 | 1.000 | 1.000 | 0.571 | 1.000 | 1.000 | 1.000 | 0.680 |
| 58 | multifurcating_6 | 1.000 | 0.731 | 1.000 | 0.385 | 0.682 | 0.690 | 1.000 |  | 0.905 |
| 59 | linear_3 | 1.000 | 1.000 | 0.521 | 1.000 | 0.396 | 0.587 | 1.000 | 1.000 | 0.473 |
| 60 | tree_1 | 1.000 | 0.584 | 1.000 | 0.312 | 0.876 | 0.888 | 0.867 | 0.887 | 0.866 |
| 61 | bifurcating_1 | 1.000 | 0.617 | 1.000 | 0.617 | 0.636 | 0.555 | 0.555 | 0.617 | 0.697 |
| 62 | binary_tree_1 | 0.906 | 0.687 | 0.875 | 0.378 | 0.908 | 0.834 | 0.722 |  | 0.831 |
| 63 | binary_tree_6 | 1.000 | 0.669 | 0.739 | 0.334 | 0.913 | 0.903 | 0.582 |  | 0.810 |
| 64 | linear_6 | 1.000 | 1.000 | 1.000 | 1.000 | 0.413 | 1.000 | 1.000 | 1.000 | 0.435 |
| 65 | bifurcating_3_2 | 1.000 | 0.629 | 1.000 | 0.629 | 0.759 | 0.631 | 0.921 | 0.629 | 0.629 |
| 66 | bifurcating_5_2 | 1.000 | 0.512 | 0.555 | 0.512 | 0.720 | 0.272 | 0.555 | 0.626 | 0.512 |
| 67 | linear_5 | 0.565 | 1.000 | 1.000 | 1.000 | 0.375 | 0.268 | 0.560 | 1.000 | 1.000 |
| 68 | linear_10 | 1.000 | 1.000 | 1.000 | 1.000 | 0.403 | 0.270 | 0.389 | 1.000 | 1.000 |
| 69 | binary_tree_4 | 0.776 | 0.738 | 0.643 | 0.377 | 0.716 | 0.524 | 0.450 | 0.848 | 0.377 |
| 70 | linear_7 | 1.000 | 1.000 | 1.000 | 1.000 | 0.572 | 0.397 | 1.000 | 1.000 | 1.000 |
| 71 | linear_1 | 1.000 | 1.000 | 1.000 | 1.000 | 0.741 | 1.000 | 1.000 | 1.000 | 1.000 |
| 72 | binary_tree_3_2 | 1.000 | 0.611 | 0.511 | 0.354 | 0.898 | 0.507 | 0.499 | 0.354 | 0.354 |
| 73 | bifurcating_10 | 1.000 | 0.983 | 1.000 | 0.604 | 0.653 | 0.272 | 1.000 | 0.604 | 0.604 |
| 74 | linear_3_2 | 1.000 | 1.000 | 1.000 | 1.000 | 0.676 | 0.397 | 1.000 | 1.000 | 1.000 |
| 75 | bifurcating_1_2 | 1.000 | 0.819 | 0.555 | 0.706 | 0.770 | 0.555 | 1.000 |  | 0.785 |
| 76 | multifurcating_1 | 1.000 | 0.510 | 0.461 | 0.335 | 0.854 | 0.861 | 0.606 | 0.509 | 0.498 |
| 77 | linear_4 | 1.000 | 1.000 | 1.000 | 1.000 | 0.371 | 0.268 | 1.000 | 0.565 | 1.000 |
| 78 | bifurcating_4_2 | 1.000 | 0.806 | 0.555 | 0.639 | 0.626 | 0.272 | 0.555 | 0.812 | 0.639 |
| 79 | bifurcating_2_2 | 1.000 | 0.681 | 0.555 | 0.681 | 0.741 | 0.272 | 0.555 | 0.753 | 0.681 |
| 80 | bifurcating_7_2 | 1.000 | 0.601 | 1.000 | 0.601 | 0.598 | 0.631 | 1.000 | 0.746 | 0.895 |
| 81 | multifurcating_3_2 | 1.000 | 0.712 | 0.319 | 0.337 | 0.878 | 0.488 | 0.511 | 0.384 | 0.507 |
|  | Average | **0.971** | 0.728 | 0.829 | 0.579 | 0.689 | 0.660 | 0.728 | 0.761 | 0.740 |

**Table 7. F1 branches of synthetic datasets**

|  |  | F1 branches | | | | | | | | |
| --- | --- | --- | --- | --- | --- | --- | --- | --- | --- | --- |
|  | dataset_name | scCRT | scTite | Slingshot | scShaper | Monocle3 | PAGA | TSCAN | scTEP | Totem |
| 1 | multifurcating_8 | 0.375 |  | 0.122 | 0.122 | 0.096 | 0.075 | 0.319 |  |  |
| 2 | bifurcating_4 | 0.778 | 0.375 | 0.761 | 0.375 | 0.710 | 0.526 | 0.772 | 0.665 | 0.706 |
| 3 | multifurcating_7 | 0.551 | 0.373 | 0.526 | 0.166 | 0.354 | 0.360 | 0.166 | 0.351 | 0.504 |
| 4 | bifurcating_8 | 0.584 |  | 0.435 | 0.435 | 0.212 | 0.447 | 0.435 |  |  |
| 5 | multifurcating_10 | 0.596 | 0.238 | 0.636 | 0.238 | 0.400 | 0.356 | 0.470 | 0.259 | 0.596 |
| 6 | tree_5 | 0.439 | 0.332 | 0.161 | 0.161 | 0.165 | 0.173 | 0.349 | 0.346 | 0.161 |
| 7 | multifurcating_4 | 0.401 | 0.288 | 0.288 | 0.288 | 0.194 | 0.251 | 0.288 | 0.288 | 0.331 |
| 8 | multifurcating_9 | 0.424 |  | 0.275 | 0.128 | 0.109 | 0.131 | 0.301 |  |  |
| 9 | binary_tree_9 | 0.583 |  | 0.621 | 0.126 | 0.315 | 0.461 | 0.485 |  |  |
| 10 | multifurcating_5 | 0.757 | 0.682 | 0.762 | 0.342 | 0.584 | 0.583 | 0.759 | 0.666 | 0.753 |
| 11 | tree_3 | 0.483 | 0.281 | 0.508 | 0.132 | 0.275 | 0.335 | 0.299 | 0.269 | 0.431 |
| 12 | linear_8 | 1.000 |  | 1.000 | 1.000 | 0.427 |  | 1.000 |  |  |
| 13 | linear_2 | 1.000 | 0.636 | 1.000 | 1.000 | 0.274 | 1.000 | 1.000 | 1.000 | 1.000 |
| 14 | linear_5 | 1.000 | 1.000 | 1.000 | 1.000 | 0.212 | 1.000 | 1.000 | 1.000 | 1.000 |
| 15 | multifurcating_3 | 0.460 | 0.319 | 0.463 | 0.106 | 0.299 | 0.264 | 0.219 | 0.369 | 0.399 |
| 16 | linear_10 | 1.000 | 1.000 | 1.000 | 1.000 | 0.435 | 0.212 | 1.000 | 1.000 | 1.000 |
| 17 | binary_tree_2 | 0.551 |  | 0.586 | 0.111 | 0.393 | 0.239 | 0.505 | 0.273 | 0.521 |
| 18 | binary_tree_4 | 0.390 | 0.203 | 0.394 | 0.118 | 0.129 | 0.154 | 0.344 | 0.176 | 0.201 |
| 19 | bifurcating_6 | 0.777 |  | 0.769 | 0.337 | 0.464 | 0.595 | 0.779 |  |  |
| 20 | tree_10 | 0.347 | 0.171 | 0.300 | 0.088 | 0.168 | 0.114 | 0.315 | 0.175 | 0.252 |
| 21 | binary_tree_7 | 0.598 | 0.468 | 0.626 | 0.102 | 0.380 | 0.413 | 0.389 | 0.289 | 0.584 |
| 22 | bifurcating_3 | 0.715 | 0.338 | 0.729 | 0.338 | 0.572 | 0.562 | 0.742 | 0.411 | 0.555 |
| 23 | linear_7 | 1.000 | 0.578 | 1.000 | 1.000 | 0.407 | 1.000 | 1.000 | 0.565 | 1.000 |
| 24 | binary_tree_8 | 0.567 |  | 0.498 | 0.163 | 0.212 | 0.407 | 0.372 |  |  |
| 25 | tree_6 | 0.469 |  | 0.592 | 0.102 | 0.330 | 0.321 | 0.385 |  |  |
| 26 | binary_tree_5 | 0.399 | 0.093 | 0.182 | 0.093 | 0.159 | 0.121 | 0.244 | 0.172 | 0.093 |
| 27 | tree_2 | 0.441 | 0.219 | 0.412 | 0.095 | 0.210 | 0.207 | 0.365 | 0.250 | 0.373 |
| 28 | linear_1 | 1.000 | 1.000 | 1.000 | 1.000 | 0.126 | 1.000 | 1.000 | 1.000 | 1.000 |
| 29 | bifurcating_2 | 0.732 | 0.384 | 0.655 | 0.371 | 0.542 | 0.310 | 0.371 | 0.371 | 0.693 |
| 30 | bifurcating_10 | 0.540 | 0.466 | 0.583 | 0.454 | 0.411 | 0.437 | 0.454 | 0.429 | 0.658 |
| 31 | multifurcating_6 | 0.552 |  | 0.536 | 0.123 | 0.303 | 0.333 | 0.334 |  |  |
| 32 | linear_3 | 0.643 | 1.000 | 1.000 | 1.000 | 0.397 | 0.317 | 1.000 | 1.000 | 1.000 |
| 33 | tree_4 | 0.612 | 0.176 | 0.601 | 0.176 | 0.186 | 0.498 | 0.439 | 0.354 | 0.570 |
| 34 | diverging_converging_1 | 0.671 | 0.349 | 0.677 | 0.177 | 0.347 | 0.436 | 0.456 | 0.395 | 0.511 |
| 35 | tree_8 | 0.380 |  | 0.256 | 0.069 | 0.156 | 0.178 | 0.259 |  |  |
| 36 | tree_7 | 0.374 |  | 0.174 | 0.127 | 0.227 | 0.080 | 0.184 | 0.144 | 0.127 |
| 37 | tree_1 | 0.449 | 0.206 | 0.477 | 0.173 | 0.184 | 0.208 | 0.173 | 0.247 | 0.173 |
| 38 | bifurcating_1 | 0.704 | 0.417 | 0.693 | 0.417 | 0.425 | 0.531 | 0.706 | 0.417 | 0.723 |
| 39 | diverging_converging_9 | 0.385 |  | 0.155 | 0.155 | 0.095 | 0.150 | 0.304 |  |  |
| 40 | bifurcating_5 | 0.647 | 0.498 | 0.337 | 0.337 | 0.278 | 0.312 | 0.635 | 0.522 | 0.327 |
| 41 | multifurcating_1 | 0.422 |  | 0.277 | 0.243 | 0.194 | 0.111 | 0.258 | 0.304 | 0.204 |
| 42 | linear_4 | 1.000 | 1.000 | 1.000 | 1.000 | 0.215 | 0.222 | 1.000 | 1.000 | 1.000 |
| 43 | binary_tree_10 | 0.531 | 0.300 | 0.602 | 0.110 | 0.233 | 0.301 | 0.504 | 0.303 | 0.415 |
| 44 | bifurcating_7 | 0.608 | 0.385 | 0.592 | 0.385 | 0.473 | 0.562 | 0.606 | 0.385 | 0.632 |
| 45 | binary_tree_1 | 0.628 | 0.138 | 0.550 | 0.110 | 0.252 | 0.277 | 0.489 | 0.280 | 0.382 |
| 46 | binary_tree_6 | 0.397 |  | 0.475 | 0.115 | 0.254 | 0.245 | 0.289 |  |  |
| 47 | bifurcating_9 | 0.726 |  | 0.742 | 0.378 | 0.244 | 0.475 | 0.732 |  |  |
| 48 | tree_9 | 0.500 |  | 0.392 | 0.134 | 0.252 | 0.282 | 0.263 |  |  |
| 49 | multifurcating_2 | 0.377 |  | 0.279 | 0.107 | 0.159 | 0.111 | 0.268 | 0.225 | 0.107 |
| 50 | linear_9 | 1.000 |  | 1.000 | 1.000 | 0.071 | 0.143 | 1.000 |  |  |
| 51 | linear_6 | 1.000 |  | 1.000 | 1.000 | 1.000 | 0.528 | 1.000 |  |  |
| 52 | tree_3 | 0.478 | 0.246 | 0.457 | 0.091 | 0.352 | 0.277 | 0.153 | 0.302 | 0.404 |
| 53 | binary_tree_3 | 0.624 | 0.646 | 0.662 | 0.142 | 0.563 | 0.494 | 0.503 | 0.562 | 0.720 |
| 54 | multifurcating_3 | 0.751 | 0.237 | 0.543 | 0.119 | 0.446 | 0.566 | 0.403 |  | 0.559 |
| 55 | bifurcating_6 | 0.921 | 0.778 | 0.946 | 0.377 | 0.646 | 0.602 | 0.510 | 0.951 | 0.858 |
| 56 | tree_6 | 0.690 | 0.504 | 0.735 | 0.136 | 0.545 | 0.472 | 0.588 |  | 0.440 |
| 57 | linear_1 | 1.000 | 1.000 | 1.000 | 1.000 | 0.395 | 1.000 | 1.000 | 1.000 | 0.454 |
| 58 | multifurcating_6 | 0.765 | 0.737 | 0.853 | 0.279 | 0.684 | 0.523 | 0.663 |  | 0.848 |
| 59 | linear_3 | 1.000 | 1.000 | 0.445 | 1.000 | 0.146 | 0.537 | 1.000 | 1.000 | 0.232 |
| 60 | tree_1 | 0.818 | 0.488 | 0.739 | 0.124 | 0.451 | 0.602 | 0.538 | 0.613 | 0.609 |
| 61 | bifurcating_1 | 0.833 | 0.387 | 0.638 | 0.433 | 0.288 | 0.433 | 0.378 | 0.382 | 0.543 |
| 62 | binary_tree_1 | 0.588 | 0.373 | 0.640 | 0.127 | 0.431 | 0.374 | 0.405 |  | 0.546 |
| 63 | binary_tree_6 | 0.699 | 0.419 | 0.503 | 0.118 | 0.590 | 0.540 | 0.235 |  | 0.552 |
| 64 | linear_6 | 1.000 | 1.000 | 1.000 | 1.000 | 0.460 | 1.000 | 1.000 | 1.000 | 0.268 |
| 65 | bifurcating_3_2 | 0.624 | 0.363 | 0.577 | 0.390 | 0.414 | 0.402 | 0.469 | 0.535 | 0.477 |
| 66 | bifurcating_5_2 | 0.376 | 0.297 | 0.320 | 0.452 | 0.184 | 0.168 | 0.309 | 0.238 | 0.452 |
| 67 | linear_5 | 0.413 | 0.539 | 0.579 | 1.000 | 0.157 | 0.144 | 0.416 | 1.000 | 0.560 |
| 68 | linear_10 | 1.000 | 0.520 | 0.521 | 1.000 | 0.144 | 0.139 | 0.312 | 0.511 | 0.533 |
| 69 | binary_tree_4 | 0.332 | 0.089 | 0.088 | 0.139 | 0.061 | 0.039 | 0.096 | 0.097 | 0.132 |
| 70 | linear_7 | 1.000 | 1.000 | 1.000 | 1.000 | 0.299 | 0.278 | 0.518 | 1.000 | 0.659 |
| 71 | linear_1 | 1.000 | 1.000 | 1.000 | 1.000 | 0.442 | 1.000 | 1.000 | 1.000 | 1.000 |
| 72 | binary_tree_3_2 | 0.501 | 0.223 | 0.308 | 0.158 | 0.306 | 0.191 | 0.229 | 0.248 | 0.253 |
| 73 | bifurcating_10 | 0.749 | 0.895 | 0.768 | 0.400 | 0.500 | 0.320 | 0.763 | 0.400 | 0.685 |
| 74 | linear_3_2 | 1.000 | 0.599 | 0.546 | 1.000 | 0.405 | 0.244 | 0.560 | 0.604 | 0.573 |
| 75 | bifurcating_1_2 | 0.721 | 0.651 | 0.404 | 0.404 | 0.623 | 0.404 | 0.602 |  | 0.686 |
| 76 | multifurcating_1 | 0.544 | 0.412 | 0.405 | 0.168 | 0.573 | 0.461 | 0.363 | 0.416 | 0.404 |
| 77 | linear_4 | 1.000 | 1.000 | 0.541 | 1.000 | 0.147 | 0.142 | 0.602 | 0.357 | 0.586 |
| 78 | bifurcating_4_2 | 0.399 | 0.235 | 0.298 | 0.406 | 0.123 | 0.157 | 0.406 | 0.250 | 0.406 |
| 79 | bifurcating_2_2 | 0.675 | 0.316 | 0.405 | 0.441 | 0.268 | 0.283 | 0.441 | 0.492 | 0.441 |
| 80 | bifurcating_7_2 | 0.774 | 0.658 | 0.712 | 0.393 | 0.487 | 0.394 | 0.715 | 0.461 | 0.961 |
| 81 | multifurcating_3_2 | 0.430 | 0.199 | 0.288 | 0.168 | 0.266 | 0.159 | 0.266 | 0.165 | 0.328 |
|  | Average | **0.658** | 0.498 | 0.588 | 0.405 | 0.332 | 0.384 | 0.524 | 0.500 | 0.541 |

**Table 8. F1 milestones of synthetic datasets**

|  |  | F1 milestones | | | | | | | | |
| --- | --- | --- | --- | --- | --- | --- | --- | --- | --- | --- |
|  | dataset_name | scCRT | scTite | Slingshot | scShaper | Monocle3 | PAGA | TSCAN | scTEP | Totem |
| 1 | multifurcating_8 | 0.402 |  | 0.215 | 0.198 | 0.108 | 0.183 | 0.329 |  |  |
| 2 | bifurcating_4 | 0.723 | 0.367 | 0.768 | 0.368 | 0.656 | 0.855 | 0.758 | 0.554 | 0.748 |
| 3 | multifurcating_7 | 0.921 | 0.390 | 0.627 | 0.293 | 0.464 | 0.792 | 0.281 | 0.363 | 0.743 |
| 4 | bifurcating_8 | 0.611 |  | 0.406 | 0.396 | 0.255 | 0.721 | 0.436 |  |  |
| 5 | multifurcating_10 | 0.887 | 0.331 | 0.850 | 0.366 | 0.429 | 0.681 | 0.548 | 0.381 | 0.727 |
| 6 | tree_5 | 0.520 | 0.315 | 0.279 | 0.274 | 0.173 | 0.331 | 0.395 | 0.272 | 0.271 |
| 7 | multifurcating_4 | 0.722 | 0.345 | 0.442 | 0.296 | 0.262 | 0.795 | 0.440 | 0.321 | 0.570 |
| 8 | multifurcating_9 | 0.435 |  | 0.290 | 0.228 | 0.124 | 0.304 | 0.313 |  |  |
| 9 | binary_tree_9 | 0.844 |  | 0.844 | 0.250 | 0.372 | 0.862 | 0.691 |  |  |
| 10 | multifurcating_5 | 0.828 | 0.561 | 0.829 | 0.453 | 0.596 | 0.919 | 0.819 | 0.497 | 0.853 |
| 11 | tree_3 | 0.664 | 0.241 | 0.631 | 0.244 | 0.329 | 0.557 | 0.361 | 0.271 | 0.564 |
| 12 | linear_8 | 0.980 |  | 0.988 | 0.961 | 0.470 |  | 0.988 |  |  |
| 13 | linear_2 | 0.913 | 0.408 | 0.948 | 0.893 | 0.405 | 1.000 | 0.948 | 0.563 | 0.481 |
| 14 | linear_5 | 0.950 | 0.921 | 0.950 | 0.915 | 0.276 | 0.928 | 0.955 | 0.929 | 0.947 |
| 15 | multifurcating_3 | 0.670 | 0.452 | 0.673 | 0.214 | 0.424 | 0.655 | 0.354 | 0.384 | 0.571 |
| 16 | linear_10 | 0.906 | 0.979 | 0.909 | 0.954 | 0.566 | 0.393 | 0.960 | 0.961 | 0.975 |
| 17 | binary_tree_2 | 0.995 |  | 0.889 | 0.215 | 0.425 | 0.592 | 0.649 | 0.296 | 0.846 |
| 18 | binary_tree_4 | 0.552 | 0.209 | 0.439 | 0.213 | 0.152 | 0.366 | 0.433 | 0.177 | 0.258 |
| 19 | bifurcating_6 | 0.812 |  | 0.829 | 0.422 | 0.474 | 0.955 | 0.795 |  |  |
| 20 | tree_10 | 0.605 | 0.187 | 0.319 | 0.179 | 0.204 | 0.267 | 0.347 | 0.222 | 0.278 |
| 21 | binary_tree_7 | 0.905 | 0.448 | 0.890 | 0.210 | 0.407 | 0.758 | 0.541 | 0.343 | 0.730 |
| 22 | bifurcating_3 | 0.858 | 0.348 | 0.858 | 0.389 | 0.597 | 0.915 | 0.796 | 0.666 | 0.659 |
| 23 | linear_7 | 0.952 | 0.722 | 0.949 | 0.901 | 0.534 | 0.903 | 0.949 | 0.705 | 0.990 |
| 24 | binary_tree_8 | 0.755 |  | 0.610 | 0.304 | 0.321 | 0.770 | 0.518 |  |  |
| 25 | tree_6 | 0.734 |  | 0.798 | 0.217 | 0.415 | 0.757 | 0.502 |  |  |
| 26 | binary_tree_5 | 0.472 | 0.173 | 0.261 | 0.177 | 0.164 | 0.272 | 0.287 | 0.176 | 0.180 |
| 27 | tree_2 | 0.689 | 0.269 | 0.721 | 0.177 | 0.267 | 0.575 | 0.636 | 0.317 | 0.489 |
| 28 | linear_1 | 0.985 | 0.972 | 0.985 | 0.983 | 0.246 | 0.962 | 0.989 | 0.972 | 0.972 |
| 29 | bifurcating_2 | 0.577 | 0.390 | 0.672 | 0.458 | 0.415 | 0.690 | 0.466 | 0.365 | 0.786 |
| 30 | bifurcating_10 | 0.545 | 0.476 | 0.812 | 0.485 | 0.394 | 0.818 | 0.453 | 0.452 | 0.814 |
| 31 | multifurcating_6 | 0.855 |  | 0.804 | 0.269 | 0.433 | 0.821 | 0.551 |  |  |
| 32 | linear_3 | 0.607 | 0.849 | 0.956 | 0.950 | 0.582 | 0.507 | 0.950 | 0.853 | 0.960 |
| 33 | tree_4 | 0.909 | 0.323 | 0.861 | 0.327 | 0.250 | 0.860 | 0.566 | 0.363 | 0.812 |
| 34 | diverging_converging_1 | 0.879 | 0.430 | 0.827 | 0.311 | 0.444 | 0.854 | 0.558 | 0.442 | 0.726 |
| 35 | tree_8 | 0.556 |  | 0.355 | 0.164 | 0.202 | 0.485 | 0.373 |  |  |
| 36 | tree_7 | 0.641 |  | 0.192 | 0.243 | 0.193 | 0.182 | 0.198 | 0.148 | 0.238 |
| 37 | tree_1 | 0.657 | 0.241 | 0.568 | 0.318 | 0.229 | 0.471 | 0.330 | 0.251 | 0.323 |
| 38 | bifurcating_1 | 0.648 | 0.408 | 0.769 | 0.432 | 0.513 | 0.802 | 0.780 | 0.409 | 0.768 |
| 39 | diverging_converging_9 | 0.437 |  | 0.323 | 0.313 | 0.118 | 0.293 | 0.337 |  |  |
| 40 | bifurcating_5 | 0.572 | 0.351 | 0.348 | 0.301 | 0.288 | 0.576 | 0.639 | 0.375 | 0.252 |
| 41 | multifurcating_1 | 0.536 |  | 0.265 | 0.299 | 0.214 | 0.269 | 0.305 | 0.238 | 0.280 |
| 42 | linear_4 | 0.742 | 0.664 | 0.856 | 0.689 | 0.285 | 0.490 | 0.946 | 0.653 | 0.703 |
| 43 | binary_tree_10 | 0.829 | 0.286 | 0.827 | 0.212 | 0.285 | 0.647 | 0.593 | 0.266 | 0.486 |
| 44 | bifurcating_7 | 0.800 | 0.250 | 0.844 | 0.414 | 0.549 | 0.869 | 0.821 | 0.352 | 0.866 |
| 45 | binary_tree_1 | 0.808 | 0.200 | 0.697 | 0.212 | 0.273 | 0.612 | 0.630 | 0.254 | 0.525 |
| 46 | binary_tree_6 | 0.641 |  | 0.614 | 0.209 | 0.332 | 0.471 | 0.328 |  |  |
| 47 | bifurcating_9 | 0.807 |  | 0.796 | 0.418 | 0.312 | 0.908 | 0.800 |  |  |
| 48 | tree_9 | 0.767 |  | 0.546 | 0.270 | 0.315 | 0.704 | 0.356 |  |  |
| 49 | multifurcating_2 | 0.659 |  | 0.250 | 0.183 | 0.234 | 0.248 | 0.261 | 0.209 | 0.157 |
| 50 | linear_9 | 0.868 |  | 0.863 | 0.771 | 0.143 | 0.326 | 0.854 |  |  |
| 51 | linear_6 | 0.975 |  | 0.984 | 0.990 | 0.989 | 0.575 | 0.988 |  |  |
| 52 | tree_3 | 0.605 | 0.227 | 0.493 | 0.149 | 0.256 | 0.426 | 0.129 | 0.247 | 0.431 |
| 53 | binary_tree_3 | 0.813 | 0.405 | 0.501 | 0.237 | 0.392 | 0.720 | 0.325 | 0.326 | 0.521 |
| 54 | multifurcating_3 | 0.659 | 0.278 | 0.423 | 0.205 | 0.399 | 0.671 | 0.286 |  | 0.526 |
| 55 | bifurcating_6 | 0.766 | 0.420 | 0.747 | 0.404 | 0.470 | 0.905 | 0.450 | 0.485 | 0.592 |
| 56 | tree_6 | 0.615 | 0.370 | 0.632 | 0.183 | 0.416 | 0.854 | 0.483 |  | 0.405 |
| 57 | linear_1 | 0.797 | 0.944 | 0.811 | 0.944 | 0.553 | 0.517 | 0.818 | 0.936 | 0.557 |
| 58 | multifurcating_6 | 0.662 | 0.455 | 0.654 | 0.284 | 0.528 | 0.825 | 0.545 |  | 0.698 |
| 59 | linear_3 | 0.836 | 0.842 | 0.542 | 0.973 | 0.237 | 0.503 | 0.846 | 0.821 | 0.355 |
| 60 | tree_1 | 0.573 | 0.335 | 0.515 | 0.170 | 0.382 | 0.620 | 0.337 | 0.425 | 0.472 |
| 61 | bifurcating_1 | 0.662 | 0.434 | 0.634 | 0.486 | 0.394 | 0.451 | 0.490 | 0.344 | 0.488 |
| 62 | binary_tree_1 | 0.702 | 0.332 | 0.594 | 0.206 | 0.351 | 0.612 | 0.313 |  | 0.450 |
| 63 | binary_tree_6 | 0.626 | 0.398 | 0.417 | 0.172 | 0.556 | 0.629 | 0.255 |  | 0.468 |
| 64 | linear_6 | 0.416 | 0.369 | 0.405 | 0.921 | 0.537 | 0.369 | 0.429 | 0.340 | 0.333 |
| 65 | bifurcating_3_2 | 0.784 | 0.350 | 0.680 | 0.418 | 0.377 | 0.745 | 0.381 | 0.366 | 0.586 |
| 66 | bifurcating_5_2 | 0.562 | 0.217 | 0.213 | 0.263 | 0.147 | 0.172 | 0.221 | 0.179 | 0.251 |
| 67 | linear_5 | 0.415 | 0.283 | 0.338 | 0.366 | 0.135 | 0.284 | 0.254 | 0.397 | 0.291 |
| 68 | linear_10 | 0.703 | 0.451 | 0.621 | 0.989 | 0.268 | 0.405 | 0.441 | 0.462 | 0.603 |
| 69 | binary_tree_4 | 0.564 | 0.082 | 0.083 | 0.117 | 0.054 | 0.078 | 0.084 | 0.088 | 0.121 |
| 70 | linear_7 | 0.960 | 0.910 | 0.961 | 0.939 | 0.344 | 0.526 | 0.629 | 0.932 | 0.728 |
| 71 | linear_1 | 0.950 | 0.912 | 0.904 | 0.961 | 0.500 | 0.714 | 0.910 | 0.951 | 0.989 |
| 72 | binary_tree_3_2 | 0.707 | 0.179 | 0.559 | 0.318 | 0.360 | 0.664 | 0.180 | 0.253 | 0.404 |
| 73 | bifurcating_10 | 0.558 | 0.551 | 0.578 | 0.473 | 0.429 | 0.840 | 0.581 | 0.479 | 0.322 |
| 74 | linear_3_2 | 0.978 | 0.576 | 0.557 | 0.973 | 0.480 | 0.538 | 0.587 | 0.535 | 0.548 |
| 75 | bifurcating_1_2 | 0.495 | 0.575 | 0.415 | 0.407 | 0.571 | 0.498 | 0.583 |  | 0.526 |
| 76 | multifurcating_1 | 0.642 | 0.329 | 0.445 | 0.300 | 0.477 | 0.741 | 0.357 | 0.321 | 0.461 |
| 77 | linear_4 | 0.592 | 0.368 | 0.320 | 0.354 | 0.118 | 0.207 | 0.310 | 0.239 | 0.292 |
| 78 | bifurcating_4_2 | 0.513 | 0.176 | 0.213 | 0.251 | 0.109 | 0.175 | 0.248 | 0.186 | 0.246 |
| 79 | bifurcating_2_2 | 0.869 | 0.325 | 0.403 | 0.506 | 0.429 | 0.662 | 0.405 | 0.280 | 0.525 |
| 80 | bifurcating_7_2 | 0.894 | 0.463 | 0.718 | 0.344 | 0.492 | 0.873 | 0.698 | 0.386 | 0.646 |
| 81 | multifurcating_3_2 | 0.809 | 0.192 | 0.426 | 0.319 | 0.316 | 0.506 | 0.227 | 0.333 | 0.518 |
|  | Average | **0.720** | 0.428 | 0.618 | 0.427 | 0.360 | 0.603 | 0.530 | 0.429 | 0.552 |

**Table 9. PCCs of synthetic datasets**

|  |  | Pseudotime PCCs | | | | | | |
| --- | --- | --- | --- | --- | --- | --- | --- | --- |
|  | dataset_name | scCRT | scTite | Slingshot | scShaper | Monocle3 | PAGA | Totm |
| 1 | multifurcating_8 | 0.791 |  | 0.862 | 0.731 | 0.889 | 0.756 |  |
| 2 | bifurcating_4 | 0.964 | 0.875 | 0.862 | 0.954 | 0.938 | 0.964 | 0.879 |
| 3 | multifurcating_7 | 0.894 | 0.657 |  | 0.795 | 0.811 | 0.781 | 0.733 |
| 4 | bifurcating_8 | 0.778 |  | 0.939 | 0.780 | 0.920 | 0.879 |  |
| 5 | multifurcating_10 | 0.902 | 0.682 |  | 0.843 | 0.793 | 0.761 | 0.615 |
| 6 | tree_5 | 0.718 | 0.750 | 0.846 | 0.722 | 0.821 | 0.832 | 0.778 |
| 7 | multifurcating_4 | 0.874 | 0.769 | 0.908 | 0.811 | 0.784 | 0.861 | 0.844 |
| 8 | multifurcating_9 | 0.845 |  | 0.834 | 0.776 | 0.872 | 0.906 |  |
| 9 | binary_tree_9 | 0.946 |  |  | 0.809 | 0.879 | 0.914 |  |
| 10 | multifurcating_5 | 0.969 | 0.896 | 0.954 | 0.226 | 0.961 | 0.984 | 0.888 |
| 11 | tree_3 | 0.917 | 0.800 |  | 0.922 | 0.928 | 0.930 | 0.836 |
| 12 | linear_8 | 0.985 |  | 0.324 | 0.995 | 0.995 |  |  |
| 13 | linear_2 | 0.334 | 0.489 | 0.979 | 0.954 | 0.946 | 0.673 | 0.632 |
| 14 | linear_5 | 0.941 | 0.846 | 0.972 | 0.962 | 0.936 | 0.954 | 0.925 |
| 15 | multifurcating_3 | 0.867 | 0.666 |  | 0.198 | 0.794 | 0.510 | 0.272 |
| 16 | linear_10 | 0.939 | 0.867 | 0.988 | 0.972 | 0.877 | 0.954 | 0.926 |
| 17 | binary_tree_2 | 0.943 |  |  | 0.607 | 0.523 | 0.764 | 0.535 |
| 18 | binary_tree_4 | 0.855 | 0.539 | 0.920 | 0.106 | 0.680 | 0.764 | 0.493 |
| 19 | bifurcating_6 | 0.968 |  |  | 0.123 | 0.969 | 0.956 |  |
| 20 | tree_10 | 0.873 | 0.674 | 0.946 | 0.845 | 0.739 | 0.874 | 0.865 |
| 21 | binary_tree_7 | 0.958 | 0.666 |  | 0.225 | 0.443 | 0.815 | 0.817 |
| 22 | bifurcating_3 | 0.969 | 0.879 |  | 0.948 | 0.879 | 0.740 | 0.779 |
| 23 | linear_7 | 0.993 | 0.530 | 0.984 | 0.989 | 0.926 | 0.941 | 0.913 |
| 24 | binary_tree_8 | 0.955 | 0.861 |  | 0.859 | 0.892 | 0.894 |  |
| 25 | tree_6 | 0.899 |  |  | 0.846 | 0.885 | 0.977 |  |
| 26 | binary_tree_5 | 0.849 | 0.901 | 0.922 | 0.917 | 0.920 | 0.942 | 0.901 |
| 27 | tree_2 | 0.852 | 0.709 |  | 0.778 | 0.775 | 0.720 | 0.610 |
| 28 | linear_1 | 0.965 | 0.831 | 0.979 | 0.979 | 0.887 | 0.867 | 0.848 |
| 29 | bifurcating_2 | 0.701 | 0.538 | 0.874 | 0.919 | 0.746 | 0.832 | 0.914 |
| 30 | bifurcating_10 | 0.913 | 0.839 | 0.957 | 0.923 | 0.884 | 0.942 | 0.953 |
| 31 | multifurcating_6 | 0.818 |  | 0.815 | 0.727 | 0.656 | 0.967 |  |
| 32 | linear_3 | 0.935 | 0.842 | 0.971 | 0.984 | 0.945 | 0.940 | 0.888 |
| 33 | tree_4 | 0.961 | 0.864 |  | 0.914 | 0.899 | 0.917 | 0.787 |
| 34 | diverging_converging_1 | 0.973 | 0.901 |  | 0.985 | 0.948 | 0.977 | 0.874 |
| 35 | tree_8 | 0.941 |  |  | 0.838 | 0.839 | 0.912 |  |
| 36 | tree_7 | 0.616 |  |  | 0.737 | 0.588 | 0.630 | 0.709 |
| 37 | tree_1 | 0.894 | 0.791 | 0.862 | 0.905 | 0.876 | 0.915 | 0.902 |
| 38 | bifurcating_1 | 0.935 | 0.838 |  | 0.972 | 0.915 | 0.966 | 0.936 |
| 39 | diverging_converging_9 | 0.838 |  | 0.735 | 0.546 | 0.689 | 0.651 |  |
| 40 | bifurcating_5 | 0.812 | 0.585 | 0.688 | 0.509 | 0.668 | 0.439 | 0.459 |
| 41 | multifurcating_1 | 0.792 |  |  | 0.650 | 0.826 | 0.406 | 0.688 |
| 42 | linear_4 | 0.470 | 0.690 | 0.857 | 0.756 | 0.892 | 0.656 | 0.764 |
| 43 | binary_tree_10 | 0.898 | 0.460 | 0.886 | 0.504 | 0.705 | 0.861 | 0.839 |
| 44 | bifurcating_7 | 0.842 | 0.011 | 0.933 | 0.037 | 0.855 | 0.925 | 0.944 |
| 45 | binary_tree_1 | 0.941 | 0.637 | 0.936 | 0.948 | 0.874 | 0.967 | 0.818 |
| 46 | binary_tree_6 | 0.854 |  |  | 0.942 | 0.856 | 0.961 |  |
| 47 | bifurcating_9 | 0.978 |  |  | 0.963 | 0.951 | 0.962 |  |
| 48 | tree_9 | 0.928 |  |  | 0.108 | 0.787 | 0.827 |  |
| 49 | multifurcating_2 | 0.823 |  | 0.824 | 0.067 | 0.567 | 0.737 | 0.791 |
| 50 | linear_9 | 0.920 |  | 0.970 | 0.942 | 0.932 | 0.943 |  |
| 51 | linear_6 | 0.970 |  | 0.995 | 0.998 | 0.996 | 0.262 |  |
| 52 | tree_3 | 0.943 | 0.638 |  | 0.567 | 0.326 | 0.821 | 0.747 |
| 53 | binary_tree_3 | 0.920 | 0.735 |  | 0.439 | 0.968 | 0.222 | 0.777 |
| 54 | multifurcating_3 | 0.878 | 0.364 |  | 0.421 | 0.731 | 0.428 |  |
| 55 | bifurcating_6 | 0.904 | 0.748 | 0.883 | 0.550 | 0.929 | 0.801 | 0.901 |
| 56 | tree_6 | 0.827 | 0.627 |  | 0.069 | 0.828 | 0.924 |  |
| 57 | linear_1 | 0.977 | 0.981 | 0.910 | 0.998 | 0.904 | 0.842 | 0.849 |
| 58 | multifurcating_6 | 0.903 | 0.778 |  | 0.508 | 0.906 | 0.883 |  |
| 59 | linear_3 | 0.817 | 0.981 | 0.919 | 0.998 | 0.622 | 0.499 | 0.940 |
| 60 | tree_1 | 0.807 | 0.498 |  | 0.170 | 0.704 | 0.633 | 0.303 |
| 61 | bifurcating_1 | 0.867 | 0.585 | 0.861 | 0.637 | 0.903 | 0.804 | 0.911 |
| 62 | binary_tree_1 | 0.747 | 0.472 | 0.512 | 0.338 | 0.717 | 0.850 |  |
| 63 | binary_tree_6 | 0.873 | 0.712 |  | 0.500 | 0.722 | 0.429 |  |
| 64 | linear_6 | 0.834 | 0.302 | 0.945 | 0.986 | 0.310 | 0.274 | 0.119 |
| 65 | bifurcating_3_2 | 0.809 | 0.173 | 0.691 | 0.393 | 0.447 | 0.435 | 0.471 |
| 66 | bifurcating_5_2 | 0.316 | 0.081 | 0.069 | 0.020 | 0.032 | 0.025 | 0.004 |
| 67 | linear_5 | 0.298 | 0.199 | 0.092 | 0.051 | 0.156 | 0.103 | 0.259 |
| 68 | linear_10 | 0.803 | 0.119 | 0.597 | 0.592 | 0.870 | 0.476 | 0.888 |
| 69 | binary_tree_4 | 0.309 | 0.017 | 0.011 | 0.014 | 0.013 | 0.036 | 0.015 |
| 70 | linear_7 | 0.925 | 0.963 | 0.899 | 0.979 | 0.989 | 0.033 | 0.939 |
| 71 | linear_1 | 0.981 | 0.979 | 0.804 | 0.996 | 0.995 | 0.989 | 0.963 |
| 72 | binary_tree_3_2 | 0.808 | 0.092 |  | 0.853 | 0.198 | 0.616 | 0.557 |
| 73 | bifurcating_10 | 0.702 | 0.963 | 0.837 | 0.758 | 0.905 | 0.908 | 0.924 |
| 74 | linear_3_2 | 0.901 | 0.102 | 0.897 | 0.857 | 0.786 | 0.900 | 0.628 |
| 75 | bifurcating_1_2 | 0.770 | 0.904 | 0.793 | 0.915 | 0.927 | 0.052 |  |
| 76 | multifurcating_1 | 0.787 | 0.633 | 0.628 | 0.119 | 0.868 | 0.656 | 0.531 |
| 77 | linear_4 | 0.118 | 0.033 |  | 0.050 | 0.025 | 0.124 | 0.020 |
| 78 | bifurcating_4_2 | 0.371 | 0.047 |  | 0.021 | 0.021 | 0.012 | 0.015 |
| 79 | bifurcating_2_2 | 0.729 | 0.446 | 0.438 | 0.983 | 0.961 | 0.951 | 0.765 |
| 80 | bifurcating_7_2 | 0.889 | 0.800 | 0.934 | 0.603 | 0.415 | 0.736 | 0.782 |
| 81 | multifurcating_3_2 | 0.764 | 0.141 |  | 0.375 | 0.540 | 0.111 | 0.407 |
|  | Average | **0.828** | 0.613 | 0.800 | 0.658 | 0.756 | 0.714 | 0.696 |
